# An immunohistochemical identification key for cell types in adult mouse prostatic and urethral tissue sections

**DOI:** 10.1371/journal.pone.0188413

**Published:** 2017-11-16

**Authors:** Kyle A. Wegner, Mark T. Cadena, Ryan Trevena, Anne E. Turco, Adam Gottschalk, Richard B. Halberg, Jinjin Guo, Jill A. McMahon, Andrew P. McMahon, Chad M. Vezina

**Affiliations:** 1 George M. O’Brien Benign Urology Center, University of Wisconsin-Madison, Madison, Wisconsin, United States of America; 2 Molecular and Environmental Toxicology Center, University of Wisconsin-Madison, Madison, Wisconsin, United States of America; 3 School of Pharmacy, University of Wisconsin-Madison, Madison, Wisconsin, United States of America; 4 School of Veterinary Medicine, University of Wisconsin-Madison, Madison, Wisconsin, United States of America; 5 Department of Oncology, McArdle Laboratory for Cancer Research, University of Wisconsin-Madison, Madison, Wisconsin, United States of America; 6 Division of Gastroenterology and Hepatology, Department of Medicine, University of Wisconsin-Madison, Madison, Wisconsin, United States of America; 7 Department of Stem Cell Biology and Regenerative Medicine, Eli and Edythe Broad-CIRM Center for Regenerative Medicine and Stem Cell Research, W.M. Keck School of Medicine of the University of Southern California, Los Angeles, CA, United States of America; 8 Carbone Cancer Center, University of Wisconsin-Madison, Madison, Wisconsin, United States of America; University of Minnesota Hormel Institute, UNITED STATES

## Abstract

Though many methods can be used to identify cell types contained in complex tissues, most require cell disaggregation and destroy information about where cells reside in relation to their microenvironment. Here, we describe a polytomous key for cell type identification in intact sections of adult mouse prostate and prostatic urethra. The key is organized as a decision tree and initiates with one round of immunostaining for nerve, epithelial, fibromuscular/hematolymphoid, or vascular associated cells. Cell identities are recursively eliminated by subsequent staining events until the remaining pool of potential cell types can be distinguished by direct comparison to other cells. We validated our identification key using wild type adult mouse prostate and urethra tissue sections and it currently resolves sixteen distinct cell populations which include three nerve fiber types as well as four epithelial, five fibromuscular/hematolymphoid, one nerve-associated, and three vascular-associated cell types. We demonstrate two uses of this novel identification methodology. We first used the identification key to characterize prostate stromal cell type changes in response to constitutive phosphatidylinositide-3-kinase activation in prostate epithelium. We then used the key to map cell lineages in a new reporter mouse strain driven by *Wnt10a*^*em1(cre/ERT2)Amc*^. The identification key facilitates rigorous and reproducible cell identification in prostate tissue sections and can be expanded to resolve additional cell types as new antibodies and other resources become available.

## Introduction

The GenitoUrinary Development Molecular Anatomy Project (GUDMAP, www.gudmap.org) is a multi-laboratory consortium dedicated to providing the scientific and medical communities with hypothesis-generating data and tools to facilitate research. A recent initiative is to build a repository of annotated genitourinary tract immunohistochemical images from *cre* expressing reporter mouse strains. The image repository will facilitate mouse strain selection by investigators, critical evaluation of research results by manuscript and grant reviewers, and generally enhance the rigor and reproducibility of *cre/lox* research studies. The most significant challenge in developing this repository is to accurately assign *cre* lineage-labels to known genitourinary cell types.

We considered multiple approaches for identifying lineage labeled cells including standard immunostaining, cell sorting, and RNA sequencing. A single round of immunostaining is a possible approach for some applications but is insufficient for comprehensive cell identification in complex tissue sections. For example, while a single round of immunostaining can be deployed to distinguish one cell type from a limited pool of closely related cells in culture (e.g. myofibroblasts from fibroblasts), the sheer diversity of cells in an intact tissue section (e.g. myofibroblasts, fibroblasts, fibrocytes, myocytes, pericytes) substantially challenges single round immunostaining for cell identification *in situ* [1,2]. Cell sorting and single cell RNASeq address the challenge of differentiating closely related cell types in complex tissues, but destroy tissue organization, cell interactions, and information about a cell’s spatial location.

We sought a comprehensive method for identifying cell types in tissue sections and were inspired by the polytomous and dichotomous identification keys used in taxonomy and phylogenetics [3]. Stepwise observations are used to systematically rule out potential cell identities until a final determination can be achieved. An identification key is diagnostic in that it can be used to distinguish a specific cell type from a broader class of cells and is differential in that it can be used to distinguish one cell from another. Immunostaining is well suited for decision making in cell identification keys because it reduces data dimensionality to a dichotomous variable: cells are either stained or unstained. We tested over 70 antibodies to identify antibody combinations (multiplexes) with the greatest power to resolve subsets of prostatic nerve fibers, epithelial cells, fibromuscular and hematolymphoid cells, and perivascular cells. We then constructed a polytomous key which organizes a series of multiplex immunostains into an optimal sequence for comprehensive cell type identification. Potential cell identities are recursively eliminated by each round of staining until cells are definitively distinguished by direct comparison with other cells. Here, we describe our mouse prostate and urethral cell identification key and provide images of identified cell types and a list of validated antibodies for multiplex immunostaining in paraffin-embedded mouse prostate tissue sections. We also demonstrate two uses of our cell identification key: objectively describing stromal cell distribution changes in a new genetically-induced mouse model of prostate cancer and identifying lineage labeled cells in a new *cre*-expressing mouse reporter strain. We anticipate this key will serve as a foundational framework for cell identification and will be broadened in the future to include additional cell types, tissues, and species.

## Materials and methods

### Mice

**Fig 1:**
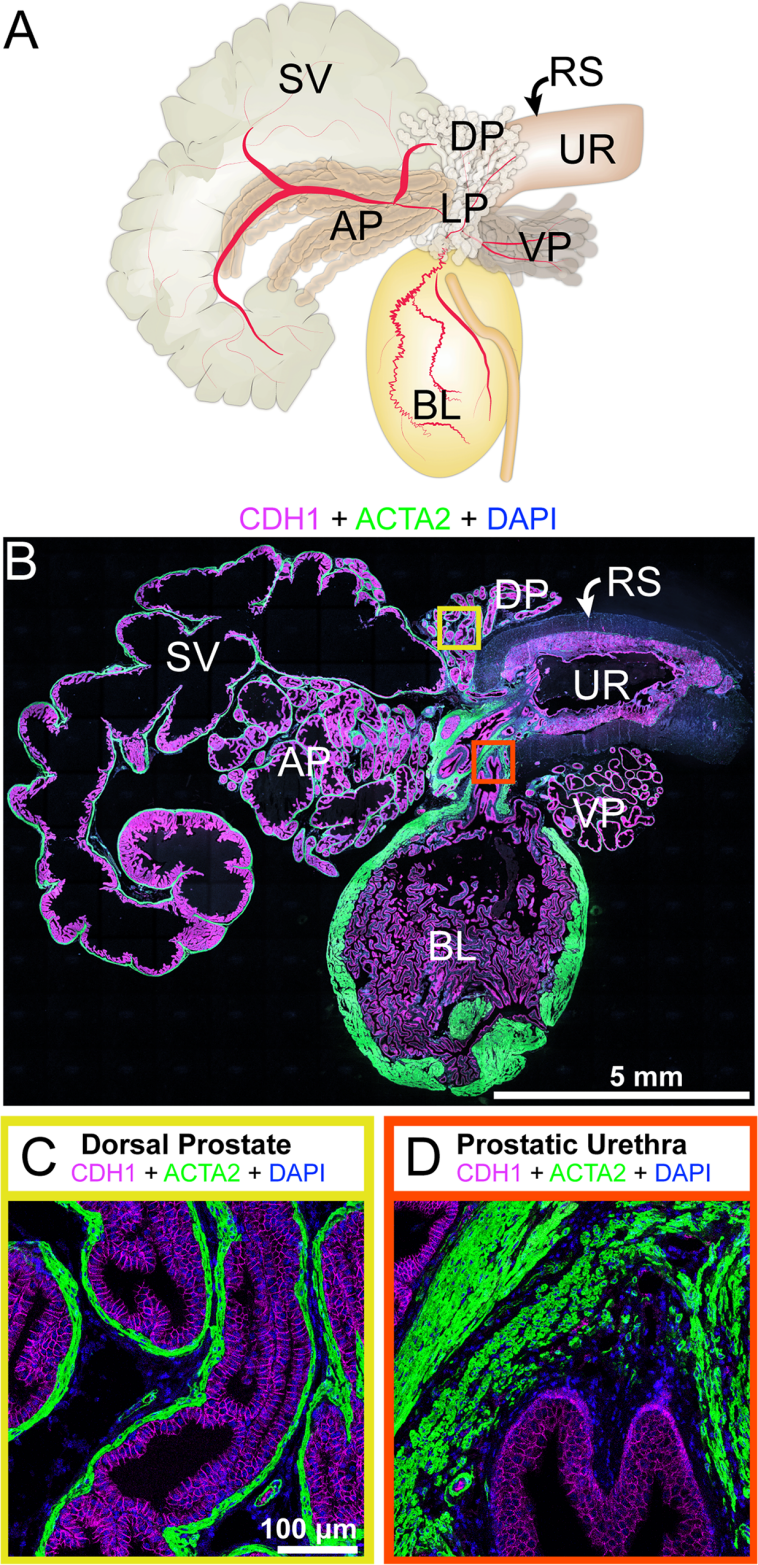
Lower urinary tract (LUT) anatomy and histology. The identification key was assembled and validated by (A) collecting LUTs from adult male mice and (B) staining paraffin sections collected from near the mid-sagittal plan. The image is a representative 5 μm LUT section immunostained with antibodies against cadherin 1 (CDH1, also known as e-cadherin, red), actin alpha 2 (ACTA2, also known as smooth muscle actin, green) and DAPI (blue). Sequential image tiles were assembled to reveal the entire lower urinary tract. Two regions of interest were captured for validation of cell types in subsequent figures: (C) the dorsal prostate external to the rhabdosphincter and (D) the prostatic urethra, located near the bladder neck and internal to the rhabdosphincter. Abbreviations are: AP, anterior prostate; BL, bladder; DP, dorsal prostate; RS, rhabdosphincter; SV, seminal vesicle; UR, pelvic urethra; VP, ventral prostate; DAPI, 2-(4-amidinophenyl)-1H -indole-6-carboxamidine.

**Fig 2:**
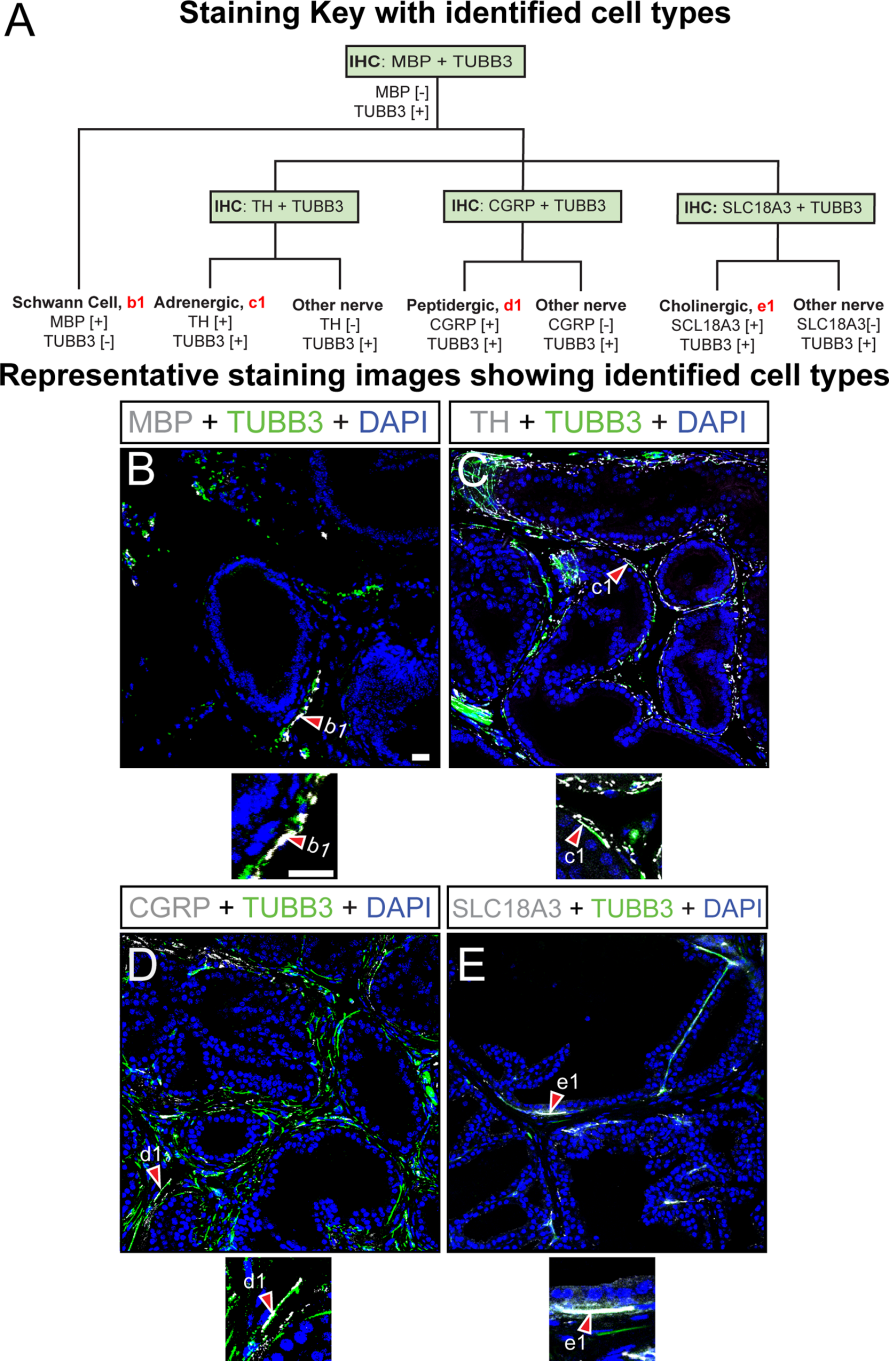
Immunohistochemical classification of neural fibers in mouse dorsal prostate. (A) Paraffin embedded adult mouse dorsal prostate sections (5 μm thickness) were stained with DAPI and antibodies against (B) MBP and TUBB3, (C) TH and TUBB3, CGRP and TUBB3, or (E) SLC18A3 and TUBB3. Identified cells include (b1) MBP1+;TUBB3- Schwann cells, (c1) TH+;TUBB3+ adrenergic fibers, (d1) CGRP+;TUBB3+ sensory fibers, and (e1) SLC18A3+;TUBB3+ cholinergic fibers. Images are representative of three mice. Abbreviations are: MBP, myelin basic protein; CGRP, calcitonin-gene-related peptide; SLC18A3, solute carrier family 18 member 3; TH, tyrosine hydroxylase; DAPI, 2-(4-amidinophenyl)-1H -indole-6-carboxamidine; Scale bar is 25 μm.

**Fig 3:**
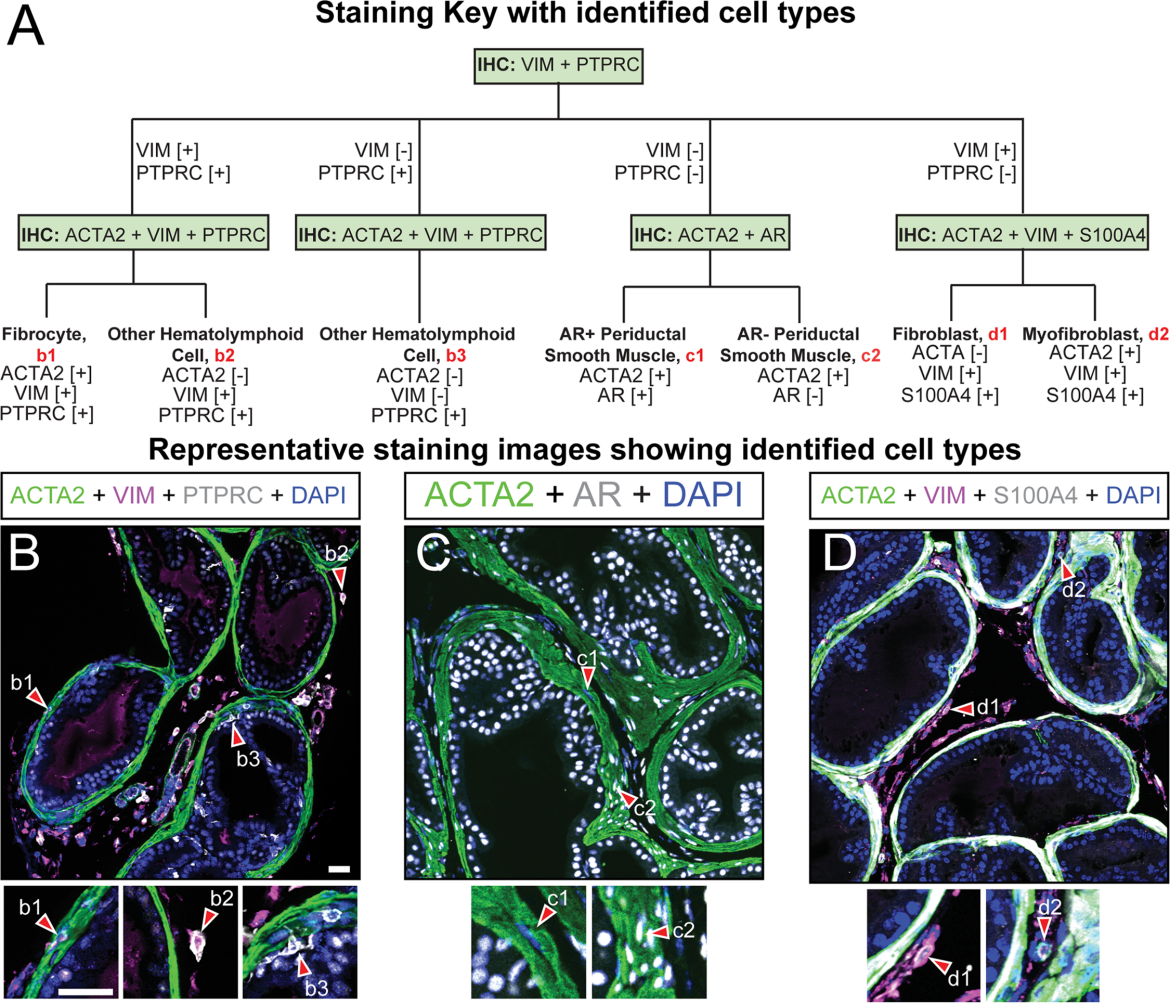
Immunohistochemical characterization of the fibromuscular components of mouse dorsal prostate. (A) Paraffin embedded adult mouse dorsal prostate sections (5 μm thickness) were stained with DAPI and antibodies against (B) ACTA2, VIM, and PTPRC, (C) ACTA2 and AR, or (D) ACTA2, VIM, and S100A4. Identified cells include (b1) ACTA2+;VIM+;PTPRC+ fibrocytes, (b2) other ACTA2-[26];VIM+;PTPRC+ (b3) or ACTA2-;VIM-;PTPRC+ hematolymphoid cells, (c1) ACTA2+;AR+ smooth muscle myocytes, (c2) ACTA2+;AR- smooth muscle myocytes, (d1) ACTA2-;VIM+;S100A4+ fibroblasts, and (d2) ACTA2+;VIM+;S100A4+ myofibroblasts Images are representative of three mice. Abbreviations: PTPRC, protein tyrosine phosphatase, receptor type, C, ACTA2, actin alpha 2; VIM, vimentin; AR, androgen receptor; S100A4, S100 calcium binding protein A4; DAPI, 2-(4-amidinophenyl)-1H -indole-6-carboxamidine; Scale bar is 25 μm.

**Fig 4:**
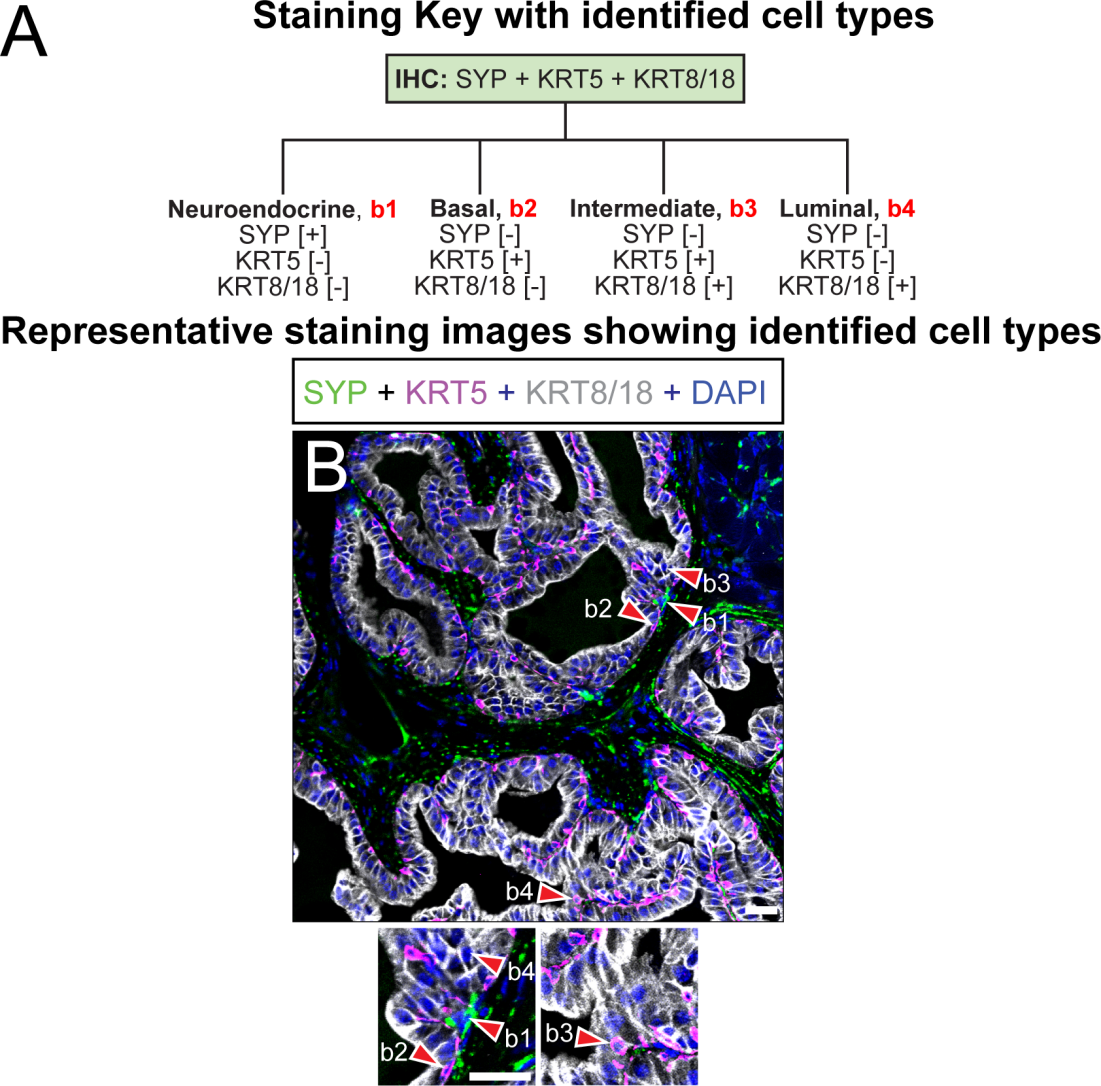
Immunohistochemical characterization of the epithelial components of mouse dorsal prostate. (A) Paraffin embedded adult mouse dorsal prostate sections (5 μm thickness) were stained with DAPI and antibodies against (B) SYP, KRT5, and KRT8/18. Identified cells include (b1) SYP+; KRT5-;KRT8/18- neuroendocrine cells, (b2) SYP-;KRT5+;KRT8/18- basal epithelial cells, (b3) SYP-; KRT5+;KRT8/18+ intermediate cells and (b4) SYP-;KRT5-;KRT8/18+ luminal epithelial cells. Images are representative of three mice. Abbreviations: SYP, synaptophysin; KRT5, keratin 5; KRT8/18, keratin 8/18; DAPI, 2-(4-amidinophenyl)-1H -indole-6-carboxamidine; Scale bar is 25 μm.

**Fig 5:**
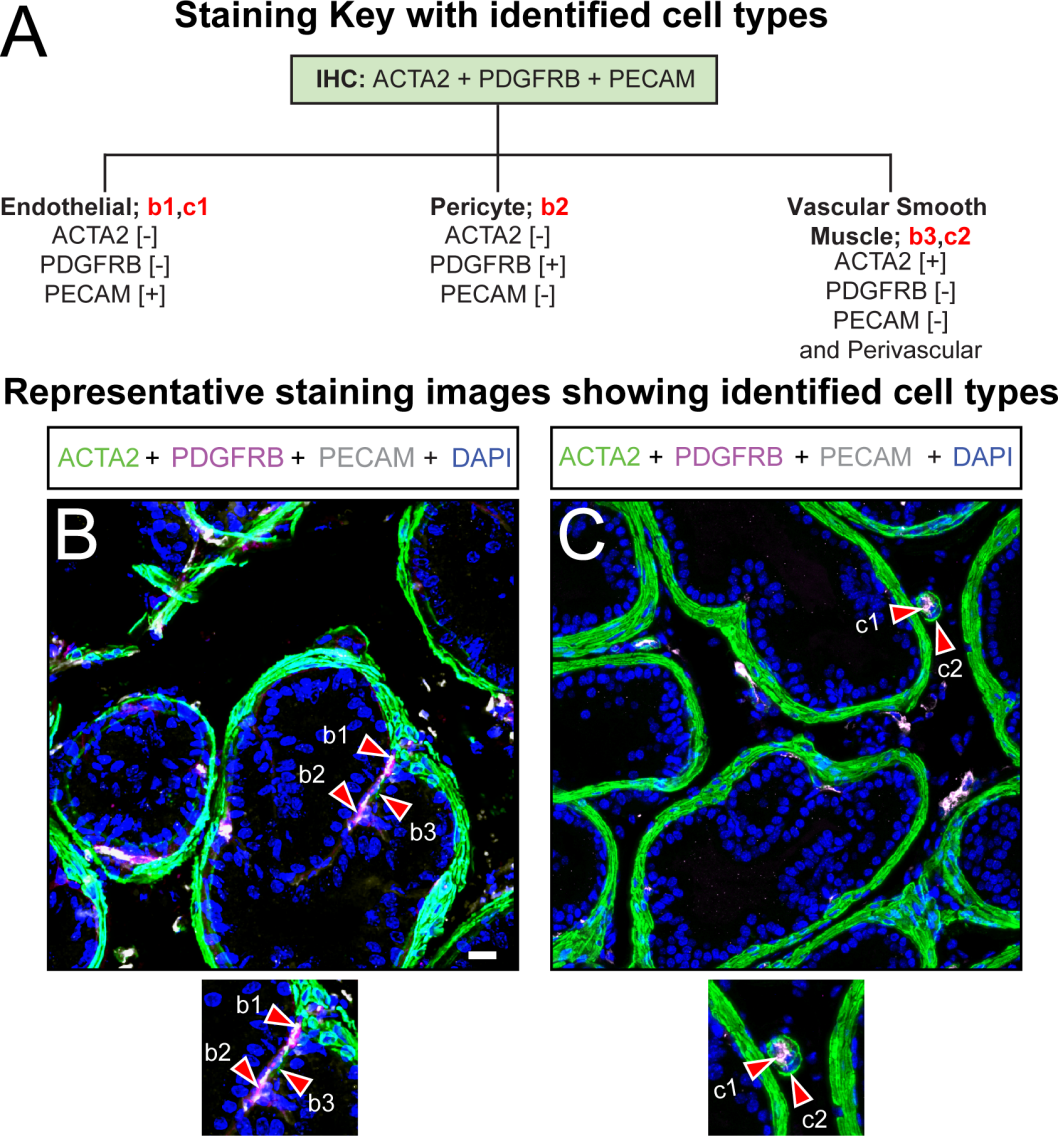
Immunohistochemical characterization of the vascular and perivascular cell types of the mouse dorsal prostate. (A) Paraffin embedded adult mouse dorsal prostate sections (15 μm thickness) were stained with DAPI and antibodies against (B, C) ACTA2, PDGFRB, and PECAM. Identified cells include (b1, c1) ACTA2-;PDGFRB-;PECAM+ endothelial cells, (b2) ACTA2-;PDGFRB+;PECAM- pericytes, and (b3, c2) ACTA2+;PDGFRB-;PECAM- vascular smooth muscle cells. Images are representative of three mice. Abbreviations are: ACTA2, actin alpha 2; PDGFRB, platelet derived growth factor receptor beta; PECAM, platelet endothelial cell adhesion molecule; DAPI, 2-(4-amidinophenyl)-1H -indole-6-carboxamidine; Scale bar is 25 μm.

**Fig 6:**
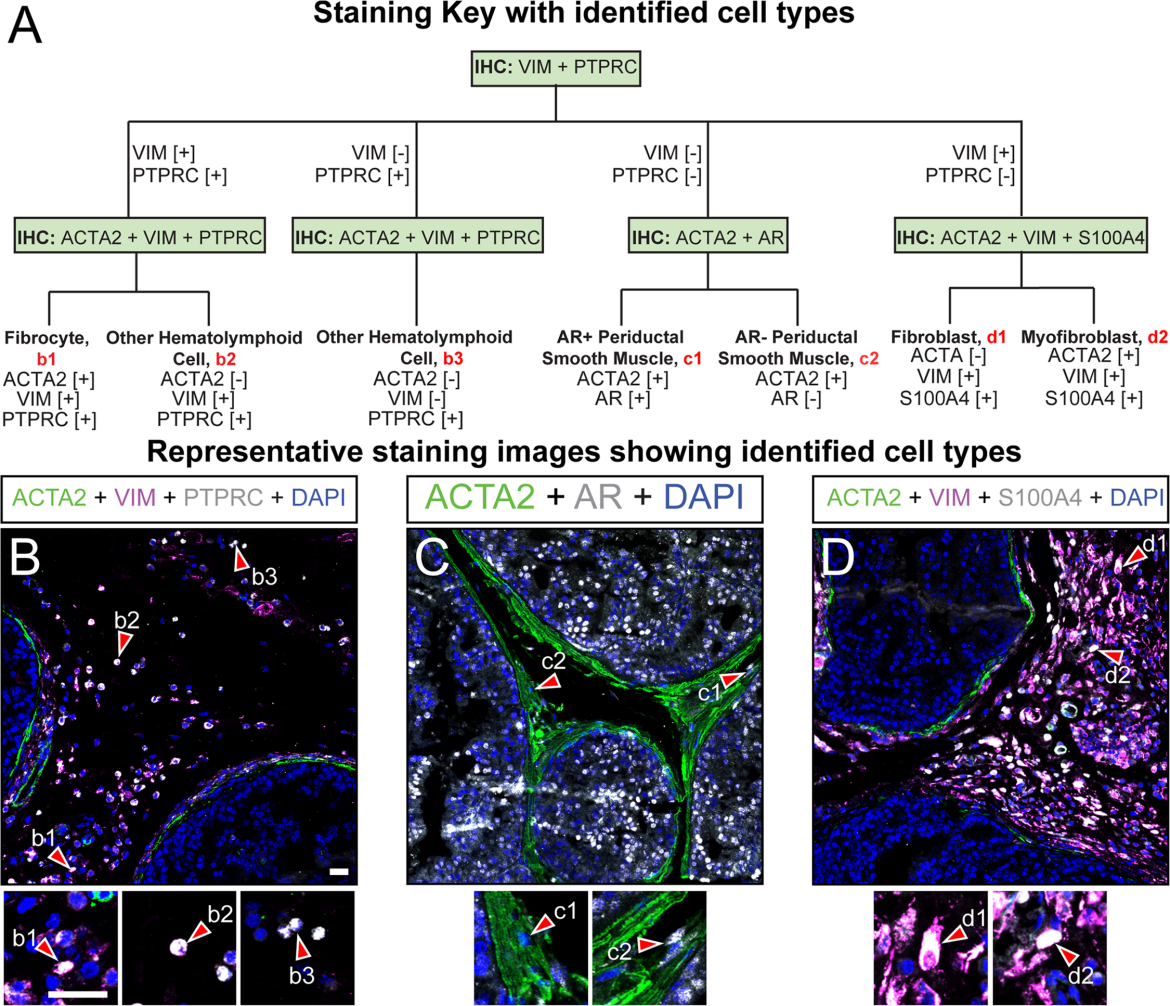
Immunohistochemical characterization of the genetically induced mouse prostate cancer. (A) Paraffin embedded adult mouse dorsal prostate sections (5 μm thickness) generated from mice with genetic activation of the PIK3/AKT signaling cascade in prostate epithelial cells (*Pbsn4*^*cre*^;PIK3ca*) and were stained with DAPI and antibodies against (B) ACTA2, VIM, and PTPRC, (C) ACTA2 and AR, or (D) ACTA2, VIM, and S100A4. Identified cells include (b1) ACTA2+;VIM+;PTPRC+ fibrocytes, (b2) other ACTA2-;VIM+;PTPRC+ (b3) or ACTA2-;VIM-;PTPRC+ hematolymphoid cells, (c1) ACTA2+;AR+ smooth muscle myocytes, (c2) ACTA2+;AR- smooth muscle myocytes, (d1) ACTA2-;VIM+;S100A4+ fibroblasts, and (d2) ACTA2+;VIM+;S100A4+ myofibroblasts. (D) Marked expansion of prostate stroma appeared to originate from expansion of the populations of putative fibroblasts. Images are representative of two mice. Abbreviations: PTPRC,CD45; ACTA2, actin alpha 2; VIM, vimentin; AR, androgen receptor; S100A4, fibroblast specific protein 1; DAPI, 2-(4-amidinophenyl)-1H -indole-6-carboxamidine; Scale bar is 25 μm.

**Fig 7:**
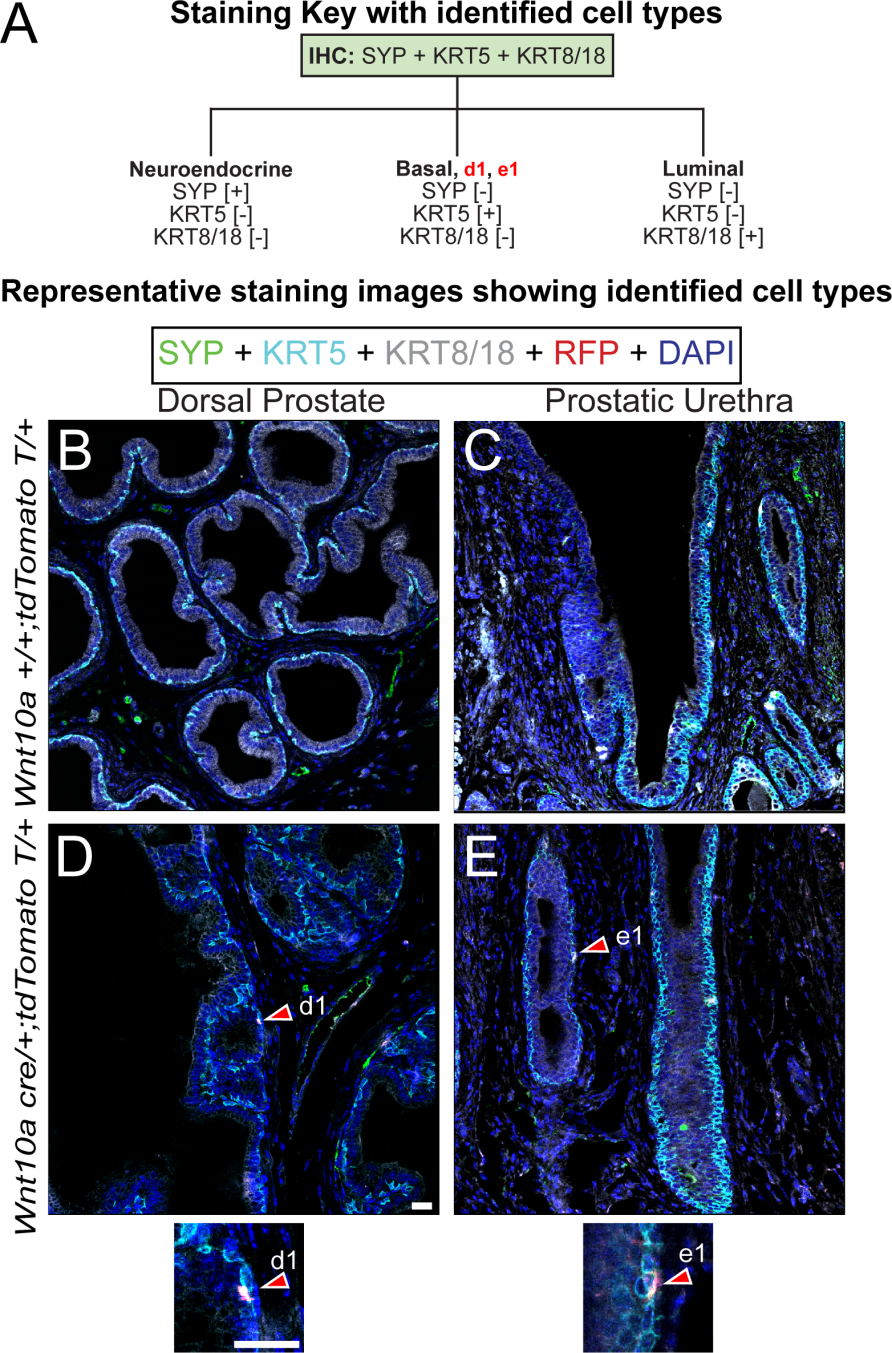
Immunohistochemical characterization of *Wnt10a*^*CreERT*^ lineage. *Wnt10a*^*CreER/+T*^;tdtomato^fl/fl^ and *Wnt10a*^*+/-*^;tdtomato^fl/fl^ (control) male mice were given a single intraperitoneal injection of tamoxifen (100 mg/kg) on postnatal day 3 and aged to two months. Prostates were sectioned (5 μm thickness), and stained with DAPI and antibodies against (B) KRT5,SYP;KRT8/18, and RFP/tdtomato. The tdtomato lineage label was identified in KRT5+;SYP-;KRT8/18- basal epithelial cells and KRT5-;SYP-;KRT8/18+ luminal epithelial cells in the (A) dorsal prostate (B) prostatic urethra of *cre* expressing mice but (C-D) not in the same regions of no *cre* control mice. Images are representative of three mice. Abbreviations: SYP, synaptophysin; KRT5, keratin 5; KRT8/18, keratin 8/18; RFP, red fluorescent protein; DAPI, 2-(4-amidinophenyl)-1H -indole-6-carboxamidine; Scale bar is 25 μm.

All procedures were approved by the University of Wisconsin Animal Care and Use Committee and conducted in accordance with the National Institutes of Health Guide for the Care and Use of Laboratory Animals. Mice were acquired from Jackson Laboratories (Bar Harbor, ME) and included several mouse strains. All images in Figs 1–5 were obtained using C57BL/6J mice (stock number 000664). Fig 6 images were from mixed background mice consisting of Tg(Pbsn-cre)4Prb/J (*Pbsn4*^*cre*^, stock number 026662), 129S1/Svlmj (stock number 002448), and C57BL/6-Gt(ROSA)26Sor^tm7(Pik3ca*,*EGFP)Rsky/J*^ (PIK3ca*, *stock number 012343*) [4,5]. Fig 7 images were from mixed background mice consisting of *C57BL/6N-Wnt10a*^*em1(cre/ERT2)Amc*^*/J* (*Wnt10a*^*creERT2*^, also known as *Wnt10a-CE*, stock number 030598) and *B6*.*Cg-Gt(ROSA)26Sor*^*tm14(CAG-tdTomato)Hze/*^*J* (*tdTomato*^*fl*^, stock number 007914) [6,7].

Genotyping was conducted as described by Jackson Laboratories. Mice were housed in Udel^®^ Polysulfone microisolator cages; the room was on 12-h light and dark cycles; room temperature was typically 20.5 ± 5°C; humidity was 30–70%. Mice were fed a 5015 Diet (PMI Nutrition International, Brentwood MO) from conception through weaning (PND 21) and an 8604 Teklad Rodent Diet thereafter (Harlan Laboratories, Madison WI). Feed and water were available *ad libitum*, and cages contained corn cob bedding. To activate *cre* in *Wnt10a*^*creERT2*^*;tdtomato*^fl/fl^ mice and their genotypic controls, male mice were given a single intraperitoneal injection of tamoxifen (100 mg/kg, Sigma #T56482, St.Louis, MO) dissolved in sterile corn oil at postnatal day 3 and prostates and urethras were evaluated two months later. Prostates and urethras were evaluated in four month old *Pbsn4*^*cre*^;PIK3ca* mice. All other tissues were evaluated at postnatal day 50. All mice were euthanized by CO_2_ asphyxiation.

### Immunohistochemistry

Tissue sections (5–15 μm thickness) were deparaffinized with xylene and rehydrated with graded ethanol. Sections for histological analysis were routinely stained with hematoxylin/eosin or fluorescent immunostaining was conducted as described previously with two modifications to the protocol [8]. A decloaking chamber (Model DC2002, Biocare Medical, Pacheco, CA) was used with either 10 mM sodium citrate (pH 6.0) or 10 mM Tris-EDTA (pH 9.5) for antigen unmasking and non-specific sites were blocked for 1 hr in TBSTw containing 1% Blocking Reagent (Roche Diagnostics, Indianapolis, IN), 5% normal donkey sera, and 1% bovine serum albumin fraction 5 (RDBTw). Antibodies are listed in S1 Table. Some tissue sections were imaged using an Eclipse E600 compound microscope (Nikon Instruments Inc., Melville, NY) fitted with a 20x dry objective (Plan Fluor NA = 0.75; Nikon, Melville, NY) and equipped with NIS elements imaging software (Nikon Instruments Inc.) Fluorescence was detected using DAPI (2-(4-amidinophenyl)-1H -indole-6-carboxamidine), FITC, Texas Red (Chroma Technology Corp, Bellows Fall, VT), and CY5 filter cubes (Nikon, Melville, NY). Other sections were imaged using an SP8 Confocal Microscope (Leica, Wetzlar, Germany) fitted with a 20x oil immersion objective (HC PL Apo CS2 NA = 0.75; Leica, Wetzlar, Germany). Samples were excited and detected using the recommended settings for each secondary antibody fluorophore. Images were captured at 1024x1024 resolution using LASX 8 software (Leica, Wetzlar, Germany). For *Pbsn4*^*cre*^;PIK3ca* mice, one sagittal section of lower urinary tract was stained and imaged from two litter independent mice. For all other studies one sagittal section of lower urinary tract was stained and imaged from each of at least three mice deriving from at least three separate litters. Representative images of dorsal prostate and ventral prostatic urethra were acquired from each image.

## Results

### Application of the identification key to mouse prostate and prostatic urethra

The male mouse genitourinary tract consists of multiple organs and the type and distribution of cells within organs differs across body axes. While a complete identification key for all parts of the genitourinary tract is beyond the scope of this study, we focus here on the pelvic portion of the mouse genitourinary tract and its urethral and prostatic constituents (Fig 1A and 1B). To enable cell visualization at single cell resolution, we concentrated on two discrete regions. We examined the dorsal prostate external to the rhabdosphincter (Fig 1C) because, in mice susceptible to prostate cancer, this region develops tumors which exhibit molecular similarities to human prostate cancer [9]. We also examined the ventral prostatic urethra internal to the rhabdosphincter (Fig 1D). This is the region where many prostatic ducts drain into the urethra and where proliferative and fibrotic pathologies have been observed in mouse models of benign prostatic hyperplasia and urinary obstruction [10].

### Nerves

The prostate and prostatic urethra contain neural fibers which transmit efferent signals to control smooth muscle contraction and prostatic secretory function, transmit afferent signals to respond to environmental stimuli, and provide trophic support to nearby non-neuronal cells [11–13]. To begin discriminating the different nerve subtypes within the prostate we applied our identification key methodology (Fig 2A). An initial stain using antibodies against myelin basic protein (MBP) and class III beta-tubulin (TUBB3) is performed. From this initial stain, putative Schwann cells (MBP+;TUBB3-) and generalized nerve fibers (MBP-;TUBB3+) are identified (Table 1). MBP+;TUBB3- cells are relatively scarce in dorsal prostate (Fig 2B), but abundant in prostatic urethra (S2B Fig). To further classify nerve fibers, subsequent immunostains with antibodies against tyrosine hydroxylase (TH), calcitonin gene related peptide (CGRP), and solute carrier family 18 (vesicular monoamine), member 3 (SLC18A3, also known as vesicular acetylcholine transporter, VaCHT) are used to create three divisions of TUBB3+ fibers. TH is considered a marker for adrenergic fibers in various tissues (Table 1). TH+;TUBB3+; fibers are abundant in dorsal prostate stroma, often encircling prostate ducts (Fig 2C). Of the three nerve divisions contained within our existing key, TH+ fibers are the most abundant in dorsal prostate and prostatic urethra. CGRP is a marker of sensory c-fibers in various tissues (Table 1) and CGRP+;TUBB3+ fibers are present throughout the stroma of the dorsal prostate (Fig 2S), but are particularly concentrated in the prostatic urethra (S2D Fig). We also observe CGRP+;TUBB3- cells in prostate epithelium (S2D Fig) and conclude these are prostate neuroendocrine cells based on their location and frequency relative to other prostate epithelial cells. CGRP has not been described previously as a marker of mouse prostatic neuroendocrine cells, but is present in bile duct and lung neuroendocrine cells [14,15]. SLC18A3 is a marker of cholinergic fibers (Table 1) and SLC18A3+;TUBB3+ fibers are rare in the dorsal prostate (Fig 2E) but quite abundant in the prostatic urethra (S2E Fig). We also observe SLC18A3+;TUBB3- cells embedded in prostatic urethral epithelium (S2E Fig) and conclude these are neuroendocrine cells based on the observation that SLC18A3 marks neuroendocrine cells in lung [16,17].

**Table 1:** Putative cell types identified using the polytomous cell identification key.

Staining Pattern	Putative cell type	References
ACTA2-;VIM+PTPRC-	Other	-
ACTA2+;VIM+PTPRC+	Fibrocyte	[18–22]
ACTA2+;AR+	AR+ Periductal Smooth Muscle Myocyte	[23–29]
ACTA2+;AR-	AR- Periductal Smooth Muscle Myocyte	
ACTA2+;VIM+;S100A4+	Myofibroblast	[24,30–32]
ACTA2-;VIM+;S100A4+	Fibroblast	[19,33–35]
ACTA2-;VIM+;PTPRC+	Other Hematolymphoid Cells	[36]
ACTA2-;VIM-;PTPRC+		
ACTA2-;VIM-;PTPRC-	Other	-
MBP+;TUBB3-	Schwann Cell	[37,38]
MBP-;TUBB3+	Nerve	-
TH+;TUBB3+	Adrenergic Nerve	[39–41]
TH-;TUBB3-	Other	-
CGRP+;TUBB3+	Sensory Nerve	[42–46]
CGRP-;TUBB3+	Other	-
SLC18A3-;TUBB3+	Cholinergic	[39,40,47]
SLC18A3-;TUBB3+	Other	-
SYP-;KRT5+;KRT8/18-	Basal Cell	[48]
SYP-;KRT5-;KRT8/18+	Luminal Cell	[48]
SYP+;KRT5-;KRT8/18-	Neuroendocrine Cell	[49,50]
SYP-;KRT5+;KRT8/18+	Intermediate Cell	(48)

### Fibromuscular and hematolymphoid cells

Prostate stroma mediates glandular development [51], prostate cancer growth [52–55] and fibrosis, the latter of which has been recently linked to urinary dysfunction [56–58]. Prostate stroma also harbors smooth muscle which can contract inappropriately and contribute to urinary symptoms in aging men [59]. Furthermore, prostate inflammation is one of the most significant predictors of lower urinary tract dysfunction in men and has been associated with chronic pelvic pain [60–63]. Pinpointing cell types responsible for behaviors that contribute to clinically significant prostate diseases will guide mechanistic studies of prostate homeostatic regulation and disease processes.

Prostatic fibromuscular and hematolymphoid cells are initially characterized by a single round of immunostaining with an antibody against protein tyrosine phosphatase, receptor type, C (PTPRC), which is present in mast cells, T cells, B cells, fibrocytes, and macrophages (Table 1), and with an antibody against vimentin (VIM), which is present in some hematolymphoid and many non hematolymphoid stromal cells (Fig 3A). Based on results of this first immunostain, additional immunostains are applied to identify specific stromal cell subtypes. We observe occasional PTPRC+ cells that are also actin alpha 2 (ACTA2) positive within the periductal smooth muscle layer, and PTPRC+ cells also rarely reside within prostate epithelium (Fig 3B). Other potential inflammatory cells, characterized by a ACTA2-;VIM+;PTPRC+ staining pattern (Table 1), are scattered throughout interductal stroma (Fig 3B). Fibrocytes have been described as cells with simultaneous expression of PTPRC, ACTA2, and vimentin (VIM) (Table 1). We observe PTPRC+ cells external to the periductal smooth muscle layer of the dorsal prostate and clustered near blood vessels (Fig 3B). A small subset of these are ACTA2+;VIM+;PTPRC+.

The smooth muscle sheath surrounding mouse prostate ducts consists almost exclusively of smooth muscle myocytes. Previous studies have found that androgen receptor (AR) expressed in the prostatic stroma mediates a significant proportion of morphological and pathological processes. During ductal morphogenesis, prostatic stromal AR is essential for epithelial cell growth. Prostatic stromal AR abundance is also important in prostate cancer progression. Prostate stromal cells begin losing AR expression during cancer progression and low AR expression in prostatic stroma is commonly found in patients who have developed resistance to androgen ablation therapy for prostate cancer [26,29]. To determine the relative abundance of AR in ACTA2+ myocytes of the adult mouse prostate performed IHC divide ACTA2+ myocytes into two subpopulations. The majority of periductal smooth muscle myocytes are ACTA2+;AR+; while ACTA2+;AR- myocytes are rare (Fig 3C, S3C Fig). This observation is consistent with previous findings in rats [28]. Whether these smooth muscle cells are functionally distinct remains to be explored.

We combine antibodies against ACTA2 with those against S100 calcium binding protein A4 (S100A4, also known as fibroblast specific protein 1, FSP1) and VIM to identify fibroblasts and myofibroblasts. Fibroblasts have been characterized as ACTA2-;VIM+;S100A4+ (Table 1). We observe cells matching this staining pattern in both dorsal prostate and prostatic urethra and they most often reside in the interductal space immediately adjacent to the periductal smooth muscle layer (Fig 3D and S3D Fig). Contractile myofibroblasts have been identified as ACTA2+;VIM+;S100A4+ (Table 1). Cells matching this expression profile are found in the interductal space but are less frequent than ACTA2-;VIM+;S100A4+; cells (Fig 3D), suggesting a relative rarity of myofibroblasts in normal prostate stroma.

### Epithelium

Prostate epithelium consists of luminal, basal, and neuroendocrine cells. Prostatic luminal epithelial cells generate most of the secreted peptides in prostatic fluid [26]. Prostatic basal cells maintain epithelial structure and integrity [64]. Although the role of prostatic neuroendocrine cells is not fully understood, neuroendocrine cells in other organs such as the intestine and lung regulate tissue growth, differentiation, and secretory activity [65].

Prostate epithelial cell identification requires staining with antibodies targeted against cytokeratins 8/18 (KRT8/18), cytokeratin 5 (KRT5), and synaptophysin (SYP) (Fig 4A). SYP-;KRT5+;KRT8/18- basal epithelial cells are concentrated on the basilar surface of prostatic ductal epithelium and distributed in an apparent discontinuous pattern (Fig 4B, S4B Fig). SYP-;KRT5-;KRT8/18+ luminal epithelial cells are polarized towards the apical surface of prostatic ductal epithelium. A very small subset of SYP-;KRT5+;Krt8/18+ intermediate cells exist in both dorsal prostate and prostatic urethral epithelium. SYP+;KRT5-;KRT8/18- prostate neuroendocrine cells are rarely observed external to the rhabdosphincter (Fig 4B), but are abundant in the prostatic urethra (S4B Fig). SYP+;KRT5-;KRT8/18- fibers (presumptive nerve fibers) are also evident in both dorsal prostate and urethral stroma (Fig 4B and S4B Fig).

### Vascular and perivascular cells

The role of angiogenesis in prostate cancer is well recognized [66] and while the role in BPH is still emerging, it is no less important. Microvessel density surrounding epithelial and stromal BPH nodules is enhanced relative to adjacent normal tissue [67] and is elevated in prostates of men with symptomatic BPH [68,69], particularly those who have failed surgical treatment [70]. Vascular smooth muscles cells regulate prostate and urethral hemodynamics, synthesize blood vessel wall components, and influence endothelial proliferation [71–73]. The role of other perivascular cells (pericytes) in adult mouse prostate is unknown, but they likely participate in angiogenesis [71,74,75] and may also contribute to pathological collagen production during organ fibrosis [76–79].

Prostate vascular and perivascular cell identification requires a multiplex stain with antibodies against ACTA2, PDGFRB, and PECAM (Fig 5A). Endothelial cells have been characterized as ACTA2-;PDGFRB-;PECAM+ (Table 1) and are present in microvessels within prostate periductal smooth muscle and interductal stroma (Fig 5B and 5C, S5B and S5C Fig). Prostatic vascular smooth muscle myocytes were described previously as ACTA2+;PDGFRB-;PECAM- (Table 1) and these cells are associated with a majority of vessels in the prostate and prostatic urethra, but are organized in a discontinuous pattern around vessels (Fig 5B and 5C and S5B Fig). Pericytes have been previously identified as ACTA2-;PDGFRB+;PECAM- (Table 1). In the dorsal prostate, pericytes are interwoven with ACTA2-;PDGFRB-;PECAM+ cells and are bounded by vascular smooth muscle myocytes and endothelial cells (Fig 5B). We do not observe pericytes in the prostatic urethra (S5B Fig).

### Using the identification key to objectively describe fibromuscular cell distribution changes during formation of genetically-induced prostate cancer

We next sought to demonstrate a utility of the cell identification key. Stromal thickening and appearance of a reactive stroma accompany many solid tumors, including prostate cancer [55]. However, the precise composition of tumor-reactive stroma and an objective characterization of how it changes *in situ* over time have not been determined. We used the identification key to determine how prostate fibromuscular cell populations change in a novel mouse model of genetically induced prostate cancer. The mouse strain models activation of PIK3/AKT signaling which accompanies many solid tumors [80], including prostate adenocarcinoma [81]. Mice expressing Cre recombinase in prostate luminal epithelial cells (*Tg(Pbsn-cre)4Prb/J*) were crossed with mice expressing a dominantly active form of the p110 catalytic subunit of phosphatidylinositol-4,5-bisphosphate 3-kinase (PI3K), resulting in offspring of a mixed genetic background (C57BL/6J x 129S1/Svlmj x FVB/NJ). Prostate sections were examined in 4 month old male mice harboring single copies of *cre* and PIK3ca* alleles (*Pbsn4*^*cre*^;PIK3ca*). Although initial observations revealed no obvious signs of epithelial hyperplasia at this age, a marked expansion of interductal stromal cell populations and loss of periductal smooth muscle integrity are evident (S7 Fig). The number of putative fibrocytes (ACTA2+,VIM+;PTPRC+) and other hematolymphoid cells (ACTA2-,VIM+;PTPRC+) appears to increase compared to wild-type controls but a majority of interductal stromal cells are ACTA2-;VIM+;PTPRC- (Fig 6B). We observed fewer periductal ACTA2+ cells in *Pbsn4*^*cre*^;PIK3ca* mice compared to controls, though the relative abundance of ACTA2+;AR+ to ACTA2+;AR- cells is similar to controls (Fig 6C). Staining for fibroblasts and myofibroblasts indicate that a majority of *Pbsn4*^*cre*^;PIK3ca* mouse interductal stromal cells are putative fibroblasts (VIM+;ACTA2-;S100A4+) and do not express ACTA2 (Fig 6D).

### Using the identification key to objectively describe lineage-labeled cells in a novel cre reporter mouse strain

A principal reason for building the prostate cell identification key is to establish an objective mechanism for characterizing *cre* expressing mouse strains. While several mouse strains have been created for genetically manipulating prostate luminal or basal epithelial cells, there is no central repository of information about the stage of development when the *cre* transgene is first expressed, whether the *cre* is expressed across all cells or evenly expressed across prostate lobes, and whether *cre* expression is the same in the distal and proximal parts of prostatic ducts. Though many studies have used *cre* expressing mouse strains to manipulate gene expression in prostate stroma, there is no centralized database describing which specific stromal cells express the *cre* and the *cre* distribution pattern. To address both of these needs, we are characterizing several *cre* expressing mouse strains, including some generated by GUDMAP, and will use the identification key for *cre* validation.

To demonstrate proof of concept, we used the identification key to validate reporter gene distribution for *Wnt10a*^*creErt2*^. We previously observed that *Wnt10a* mRNA is selectively expressed in a subset of basal epithelial cells within the female and male mouse urogenital sinus, including prostatic ductal bud epithelium in males [82]. This *Wnt10a*^*creErt2*^ mouse strain was generated to enable genetic manipulation of this epithelial subset. To identify the cell types deriving from this epithelial cell population, Cre was activated in postnatal day 3 male mice (*Wnt10a*^*creErt2*^*;tdtomato*^*fl/fl*^) by administering a single i.p. dose of tamoxifen and tdtomato reporter expression was mapped to prostate epithelial cell types when the mice reached 50 days of age. Tdtomato positive cells are infrequent and are mapped to KRT8/18-;KRT5+;SYP- (basal) and KRT8/18+;KRT5-;SYP- (luminal) epithelial cells of the dorsal prostate and prostatic urethra (Fig 7). Tdtomato is not observed in any KRT8/18+-KRT5-;SYP+ (neuroendocrine) cells of the prostate or prostatic urethra.

## Discussion

Here we described a polytomous key for immunohistochemical identification of cell types in adult mouse prostate and prostatic urethra. The key currently identifies sixteen unique cell types and some of its many uses include objectively defining lineage labeled cells, proliferating cells, or changes in cellular distribution in response to aging, inflammation, benign hyperplasia, cancer, or environmental exposures.

We created the mouse prostate cell identification key with the expectation it would expand to accommodate additional cell types as knowledge about prostate cellular composition is advanced by RNASeq, flow cytometry, and other methods. For example, this study utilized PTPRC as a biomarker of cells deriving from a hematolymphoid lineage and we multiplexed PTPRC with ACTA2 and VIM antibodies to identify putative fibrocytes, which derive from hematolymphoid progenitors. Though our initial version of this key does not further resolve hematolymphoid cell types, additional branches can be incorporated into future versions to account for the more than 14 different monoclonal antibodies used to differentiate hematolymphoid cell types [83].

A future opportunity afforded by this key is to objectively characterize changes in prostatic cellular composition during prostate glandular development, response to androgen deprivation, or during regeneration of the castrated prostate. The key is ideally paired with genetic lineage tracing methods that incorporate an indelible label into a cell and its daughter cells. For example, we determined that *Wnt10a* expressing cells in the postnatal day 5 prostate give rise to a limited subset of prostatic basal and luminal epithelial cells in the adult mouse but do not appear to vigorously expand and thus likely have limited progenitor activity.

We also used our key to characterize prostate stromal cell distribution changes in mice expressing a constitutively active form of PI3K in prostate epithelial cells. We observed formation of a new stromal microenvironment that closely resembles the reactive stroma observed near human cancer tumors. In many human cancers, including prostate, a major cellular component of the reactive stroma are myofibroblasts [84]. To differentiate myofibroblasts from smooth muscle cells in prostate stroma, earlier studies used complementary but not sequential IHC. These studies found that human prostate cancer is characterized by increased percentages of VIM+ cells, with no change in ACTA2 positivity [85]. Our results expand on previous findings by using a wider panel of antibodies to examine the reactive stroma with single cell resolution and found that in Pbsn4cre;PIK3ca* mice, putative fibroblasts (S100A4+;VIM+;ACTA2-) dominate the reactive stroma. Although a majority of the cells within the prostate stroma of Pbsn4cre;PIK3ca* mice are ACTA2- and likely not myofibroblasts, the potential for the fibroblasts present in the stroma to undergo phenoconversion to a myofibroblast-like phenotype and begin producing ACTA2 as disease progresses is highly plausible. Also, because the stromal response to pathologies such as cancer differs among mouse strains [86–88], investigators can use our key to compare the stromal cellular makeup among strains and at differing spatial locations with respect to sources of inflammation or tumor boundaries. Similar comparisons can be made between or among species. Although initially validated for mouse prostate, many of the antibodies used in our key are advertised to work in multiple species and organ systems and validating them across species is a future goal.

## Supporting information

S1 Fig Complete identification key. (TIF)

S2 Fig Immunohistochemical classification of neural fibers in mouse prostatic urethra. (A) Paraffin embedded adult mouse prostatic urethra sections (5 μm thickness) were stained with DAPI and antibodies against (B) MBP and TUBB3 (C) TH and TUBB3, CGRP and TUBB3, or (E) SLC18A3 and TUBB3. Identified cells include (b1) MBP1+; TUBB3-; Schwann cells, (c1) TH+;TUBB3+ adrenergic fibers (d1) CGRP+;TUBB3+ sensory fibers, (e1) SLC18A3+;TUBB3+ cholinergic fibers. Images are representative of three mice. Abbreviations are: MBP, myelin basic protein; CGRP, calcitonin-gene-related peptide; SLC18A3, solute carrier family 18 member 3; TH, tyrosine hydroxylase; DAPI, 2-(4-amidinophenyl)-1H -indole-6-carboxamidine; Scale bar is 25 μm.
(TIF)

S3 Fig Immunohistochemical characterization of the fibromuscular components of mouse prostatic urethra. (A) Paraffin embedded adult mouse prostatic urethra sections (5 μm thickness) were stained with DAPI and antibodies against (B) ACTA2, VIM, and PTPRC, (C) ACTA2 and AR, or (D) ACTA2, VIM, and S100A4. The identified cells include (b1) ACTA2-;VIM+;PTPRC+ hematolymphoid cells, (c1) ACTA2+;AR+ smooth muscle myoctyes, (c2) ACTA2+;AR- smooth muscle myocytes, (d1) ACTA2-;VIM+;S100A4+ fibroblasts, and (d2) ACTA2+;VIM+;S100A4+ myofibroblasts Images are representative of n = 3 mice. Abbreviations: PTPRC, CD45; ACTA2, actin alpha 2; VIM, vimentin; AR, androgen receptor; S100A4, fibroblast specific protein 1; DAPI, 2-(4-amidinophenyl)-1H -indole-6-carboxamidine; Scale bar is 25 μm.
(TIF)

S4 Fig Immunohistochemical characterization of the epithelial components of mouse prostatic urethra. (A) Paraffin embedded adult mouse prostatic urethra sections (5 μm thickness) were stained with DAPI and antibodies against (B) KRT5, SYP, and KRT8/18. Identified cells include (b1) KRT5-;SYP+;KRT8/18- neuroendocrine cells, (b2) KRT5+;SYP-;KRT8/18- basal epithelial cells, and (b3) KRT5-;SYP-;KRT8/18+ luminal epithelial cells. Images are representative of three mice. Abbreviations: SYP, synaptophysin; KRT5, keratin 5; KRT8/18, keratin 8/18; DAPI, 2-(4-amidinophenyl)-1H -indole-6-carboxamidine; Scale bar is 25 μm.
(TIF)

S5 Fig Immunohistochemical characterization of the vascular and perivascular cell types of the mouse prostatic urethra. (A) Paraffin embedded adult mouse prostatic urethra sections (15 μm thickness) were stained with DAPI and antibodies against (B, C) ACTA2, PDGFRB, and PECAM. Identified cells include (b1, c1) ACTA2-;PDGFRB-;PECAM+ endothelial cells, (b2) ACTA2-;PDGFRB+;PECAM- pericytes, and (b3, c2) ACTA2+;PDGFRB-;PECAM- vascular smooth muscle cells. Images are representative of three mice. Abbreviations: ACTA2, actin alpha 2; PDGFRB, platelet derived growth factor receptor beta; PECAM, platelet endothelial cell adhesion molecule; DAPI, 2-(4-amidinophenyl)-1H -indole-6-carboxamidine; Scale bar is 25 μm.
(TIF)

S6 Fig Immunohistochemical characterization of *Wnt10a*^*CreERT*^ lineage in mouse prostate luminal epithelial cells. *Wnt10a*^*CreER/+T*^;tdtomato^fl/fl^ and *Wnt10a*^*+/-*^;tdtomato^fl/fl^ (control) male mice were given a single intraperitoneal injection of tamoxifen (100 mg/kg) on postnatal day 3 and aged to two months. Prostates were sectioned (5 μm thickness), and stained with DAPI and antibodies against (A) CDH1, and RFP/tdtomato. The tdtomato lineage label was identified in CDH1+ luminal epithelial cells. Image is representative of three mice. Abbreviations: CDH1, E Cadherin; RFP, red fluorescent protein; DAPI, 2-(4-amidinophenyl)-1H -indole-6-carboxamidine; Scale bar is 25 μm.
(TIF)

S7 Fig Expansion of prostatic stroma in genetically induced mouse prostate cancer model. (A) Paraffin embedded adult mouse prostate sections (5 μm thickness) generated from mice with genetic activation of the PIK3/AKT signaling cascade in prostate epithelial cells (*Pbsn4*^*cre*^;PIK3ca*) and were stained with hematoxylin and eosin to reveal a marked increase in the fibromuscular stroma of the prostate. Scale bar is 100 μm.
(TIF)

S1 Table Antibodies used for immunostaining. (DOCX)

## References

[1] Herum KM, Choppe J, Kumar A, Engler AJ, McCulloch AD (2017) Mechanical regulation of cardiac fibroblast profibrotic phenotypes. Mol Biol Cell 28: 1871–1882. doi: 10.1091/mbc.E17-01-0014 28468977 10.1091/mbc.E17-01-0014 PMC5541838

[2] Untergasser G, Gander R, Lilg C, Lepperdinger G, Plas E, Berger P. (2005) Profiling molecular targets of TGF-beta1 in prostate fibroblast-to-myofibroblast transdifferentiation. Mech Ageing Dev 126: 59–69. doi: 10.1016/j.mad.2004.09.023 15610763 10.1016/j.mad.2004.09.023

[3] Barré P, Stöver BC, Müller KF, Steinhage V (2017) LeafNet: A computer vision system for automatic plant species identification. Ecological Informatics.

[4] Srinivasan L, Sasaki Y, Calado DP, Zhang B, Paik JH, DePinho RA, et al (2009) PI3 kinase signals BCR-dependent mature B cell survival. Cell 139: 573–586. doi: 10.1016/j.cell.2009.08.041 19879843 10.1016/j.cell.2009.08.041 PMC2787092

[5] Wu X, Wu J, Huang J, Powell WC, Zhang J, Matusik RJ, et al (2001) Generation of a prostate epithelial cell-specific Cre transgenic mouse model for tissue-specific gene ablation. Mech Dev 101: 61–69. 11231059 10.1016/s0925-4773(00)00551-7

[6] McMahon A, Zhang, P. and colleagues (2010) The GenitoUrinary Development Molecular Anatomy Project (GUDMAP). 2010. Summary of mouse strains characterized by GUDMAP consortium.

[7] Madisen L, Zwingman TA, Sunkin SM, Oh SW, Zariwala HA, Gu H, et al (2010) A robust and high-throughput Cre reporting and characterization system for the whole mouse brain. Nat Neurosci 13: 133–140. doi: 10.1038/nn.2467 20023653 10.1038/nn.2467 PMC2840225

[8] Abler LL, Keil KP, Mehta V, Joshi PS, Schmitz CT, Vezina CM. (2011) A high-resolution molecular atlas of the fetal mouse lower urogenital tract. Dev Dyn 240: 2364–2377. doi: 10.1002/dvdy.22730 21905163 10.1002/dvdy.22730 PMC3583531

[9] Thielen JL, Volzing KG, Collier LS, Green LE, Largaespada DA, Marker PC. (2007) Markers of prostate region-specific epithelial identity define anatomical locations in the mouse prostate that are molecularly similar to human prostate cancers. Differentiation 75: 49–61. doi: 10.1111/j.1432-0436.2006.00115.x 17244021 10.1111/j.1432-0436.2006.00115.x

[10] Nicholson TM, Ricke EA, Marker PC, Miano JM, Mayer RD, Timms BG, et al (2012) Testosterone and 17beta-estradiol induce glandular prostatic growth, bladder outlet obstruction, and voiding dysfunction in male mice. Endocrinology 153: 5556–5565. doi: 10.1210/en.2012-1522 22948219 10.1210/en.2012-1522 PMC3473198

[11] Fromont G, Godet J, Pires C, Yacoub M, Dore B, Irani J. (2012) Biological significance of perineural invasion (PNI) in prostate cancer. Prostate 72: 542–548. doi: 10.1002/pros.21456 21748758 10.1002/pros.21456

[12] Lee S, Yang G, Xiang W, Bushman W (2016) Retrograde double-labeling demonstrates convergent afferent innervation of the prostate and bladder. Prostate. 10.1002/pros.23170 26939943

[13] Schwartz ES, La JH, Young EE, Feng B, Joyce S, Gebhart GF. (2016) Chronic Prostatitis Induces Bladder Hypersensitivity and Sensitizes Bladder Afferents in the Mouse. J Urol 196: 892–901. doi: 10.1016/j.juro.2016.03.077 26997315 10.1016/j.juro.2016.03.077 PMC5283779

[14] Glaser S, Gaudio E, Renzi A, Mancinelli R, Ueno Y, Venter J, et al (2011) Knockout of the neurokinin-1 receptor reduces cholangiocyte proliferation in bile duct-ligated mice. Am J Physiol Gastrointest Liver Physiol 301: G297–305. doi: 10.1152/ajpgi.00418.2010 21596993 10.1152/ajpgi.00418.2010 PMC3154601

[15] Gu X, Karp PH, Brody SL, Pierce RA, Welsh MJ, Holtzman MJ, et al (2014) Chemosensory functions for pulmonary neuroendocrine cells. Am J Respir Cell Mol Biol 50: 637–646. doi: 10.1165/rcmb.2013-0199OC 24134460 10.1165/rcmb.2013-0199OC PMC4068934

[16] Brouns I, Oztay F, Pintelon I, De Proost I, Lembrechts R, Timmermans JP, et al (2009) Neurochemical pattern of the complex innervation of neuroepithelial bodies in mouse lungs. Histochem Cell Biol 131: 55–74. doi: 10.1007/s00418-008-0495-7 18762965 10.1007/s00418-008-0495-7

[17] Kummer W, Lips KS, Pfeil U (2008) The epithelial cholinergic system of the airways. Histochem Cell Biol 130: 219–234. doi: 10.1007/s00418-008-0455-2 18566825 10.1007/s00418-008-0455-2 PMC2491704

[18] Reilkoff RA, Bucala R, Herzog EL (2011) Fibrocytes: emerging effector cells in chronic inflammation. Nat Rev Immunol 11: 427–435. doi: 10.1038/nri2990 21597472 10.1038/nri2990 PMC3599774

[19] Pilling D, Fan T, Huang D, Kaul B, Gomer RH (2009) Identification of markers that distinguish monocyte-derived fibrocytes from monocytes, macrophages, and fibroblasts. PLoS One 4: e7475 doi: 10.1371/journal.pone.0007475 19834619 10.1371/journal.pone.0007475 PMC2759556

[20] Abu El-Asrar AM, Struyf S, Van Damme J, Geboes K (2008) Circulating fibrocytes contribute to the myofibroblast population in proliferative vitreoretinopathy epiretinal membranes. Br J Ophthalmol 92: 699–704. doi: 10.1136/bjo.2007.134346 18441176 10.1136/bjo.2007.134346

[21] Wong L, Hutson PR, Bushman W (2014) Prostatic inflammation induces fibrosis in a mouse model of chronic bacterial infection. PLoS One 9: e100770 doi: 10.1371/journal.pone.0100770 24950301 10.1371/journal.pone.0100770 PMC4065064

[22] Bucala R, Spiegel LA, Chesney J, Hogan M, Cerami A (1994) Circulating fibrocytes define a new leukocyte subpopulation that mediates tissue repair. Mol Med 1: 71–81. 8790603 PMC2229929

[23] Owens GK, Kumar MS, Wamhoff BR (2004) Molecular regulation of vascular smooth muscle cell differentiation in development and disease. Physiol Rev 84: 767–801. doi: 10.1152/physrev.00041.2003 15269336 10.1152/physrev.00041.2003

[24] Council L, Hameed O (2009) Differential expression of immunohistochemical markers in bladder smooth muscle and myofibroblasts, and the potential utility of desmin, smoothelin, and vimentin in staging of bladder carcinoma. Mod Pathol 22: 639–650. doi: 10.1038/modpathol.2009.9 19252475 10.1038/modpathol.2009.9

[25] Taboga SR, Scortegagna E, Siviero MP, Carvalho HF (2008) Anatomy of smooth muscle cells in nonmalignant and malignant human prostate tissue. Anat Rec (Hoboken) 291: 1115–1123. 18727055 10.1002/ar.20728

[26] Marker PC, Donjacour AA, Dahiya R, Cunha GR (2003) Hormonal, cellular, and molecular control of prostatic development. Dev Biol 253: 165–174. 12645922 10.1016/s0012-1606(02)00031-3

[27] Hayward SW, Cunha GR (2000) The prostate: development and physiology. Radiol Clin North Am 38: 1–14. 10664663 10.1016/s0033-8389(05)70146-9

[28] Hayward SW, Baskin LS, Haughney PC, Foster BA, Cunha AR, Dahiya R, et al (1996) Stromal development in the ventral prostate, anterior prostate and seminal vesicle of the rat. Acta Anat (Basel) 155: 94–103. 8828707 10.1159/000147794

[29] Singh M, Jha R, Melamed J, Shapiro E, Hayward SW, Lee P. (2014) Stromal androgen receptor in prostate development and cancer. Am J Pathol 184: 2598–2607. doi: 10.1016/j.ajpath.2014.06.022 25088980 10.1016/j.ajpath.2014.06.022 PMC4188859

[30] Masur SK, Dewal HS, Dinh TT, Erenburg I, Petridou S (1996) Myofibroblasts differentiate from fibroblasts when plated at low density. Proc Natl Acad Sci U S A 93: 4219–4223. 8633044 10.1073/pnas.93.9.4219 PMC39515

[31] Brenner DA, Kisseleva T, Scholten D, Paik YH, Iwaisako K, Inokuchi S, et al (2012) Origin of myofibroblasts in liver fibrosis. Fibrogenesis Tissue Repair 5: S17 doi: 10.1186/1755-1536-5-S1-S17 23259769 10.1186/1755-1536-5-S1-S17 PMC3368775

[32] Schmitt-Graff A, Desmouliere A, Gabbiani G (1994) Heterogeneity of myofibroblast phenotypic features: an example of fibroblastic cell plasticity. Virchows Arch 425: 3–24. 7921410 10.1007/BF00193944

[33] Lawson WE, Polosukhin VV, Zoia O, Stathopoulos GT, Han W, Plieth D, et al (2005) Characterization of fibroblast-specific protein 1 in pulmonary fibrosis. Am J Respir Crit Care Med 171: 899–907. doi: 10.1164/rccm.200311-1535OC 15618458 10.1164/rccm.200311-1535OC

[34] Strutz F, Okada H, Lo CW, Danoff T, Carone RL, Tomaszewski JE, et al (1995) Identification and characterization of a fibroblast marker: FSP1. J Cell Biol 130: 393–405. 7615639 10.1083/jcb.130.2.393 PMC2199940

[35] Camelliti P, Green CR, LeGrice I, Kohl P (2004) Fibroblast network in rabbit sinoatrial node: structural and functional identification of homogeneous and heterogeneous cell coupling. Circ Res 94: 828–835. doi: 10.1161/01.RES.0000122382.19400.14 14976125 10.1161/01.RES.0000122382.19400.14

[36] Rehg JE, Bush D, Ward JM (2012) The utility of immunohistochemistry for the identification of hematopoietic and lymphoid cells in normal tissues and interpretation of proliferative and inflammatory lesions of mice and rats. Toxicol Pathol 40: 345–374. doi: 10.1177/0192623311430695 22434870 10.1177/0192623311430695

[37] Marty MC, Alliot F, Rutin J, Fritz R, Trisler D, Pessac B. (2002) The myelin basic protein gene is expressed in differentiated blood cell lineages and in hemopoietic progenitors. Proc Natl Acad Sci U S A 99: 8856–8861. doi: 10.1073/pnas.122079599 12084930 10.1073/pnas.122079599 PMC124388

[38] Lehmann HC, Chen W, Mi R, Wang S, Liu Y, Rao M, et al (2012) Human Schwann cells retain essential phenotype characteristics after immortalization. Stem Cells Dev 21: 423–431. doi: 10.1089/scd.2010.0513 21585251 10.1089/scd.2010.0513 PMC3272243

[39] Zaitouna M, Alsaid B, Lebacle C, Timoh KN, Benoit G, Bessede T. (2017) Origin and nature of pelvic ureter innervation. Neurourol Urodyn 36: 271–279. doi: 10.1002/nau.22919 28235166 10.1002/nau.22919

[40] Creze M, Zaitouna M, Krystel NT, Diallo D, Lebacle C, Bellin M, et al (2016) Functional and structural microanatomy of the fetal sciatic nerve. Muscle Nerve. 10.1002/mus.25531 28006841

[41] Ohki K, Ohno Y, Suzuki K (2010) The investigation of ureteral sympathetic innervation, using semi-serial sections: why does the alpha1-adrenergic receptor antagonist work well for ureteral stones? Int Urol Nephrol 42: 113–117. doi: 10.1007/s11255-009-9592-3 19551485 10.1007/s11255-009-9592-3

[42] Peleshok JC, Ribeiro-da-Silva A (2011) Delayed reinnervation by nonpeptidergic nociceptive afferents of the glabrous skin of the rat hindpaw in a neuropathic pain model. J Comp Neurol 519: 49–63. doi: 10.1002/cne.22500 21120927 10.1002/cne.22500

[43] Saeed AW, Pawlowski SA, Ribeiro-da-Silva A (2015) Limited changes in spinal lamina I dorsal horn neurons following the cytotoxic ablation of non-peptidergic C-fibers. Mol Pain 11: 54 doi: 10.1186/s12990-015-0060-z 26353788 10.1186/s12990-015-0060-z PMC4564961

[44] Magnussen C, Hung SP, Ribeiro-da-Silva A (2015) Novel expression pattern of neuropeptide Y immunoreactivity in the peripheral nervous system in a rat model of neuropathic pain. Mol Pain 11: 31 doi: 10.1186/s12990-015-0029-y 26012590 10.1186/s12990-015-0029-y PMC4449610

[45] Shelukhina I, Paddenberg R, Kummer W, Tsetlin V (2015) Functional expression and axonal transport of alpha7 nAChRs by peptidergic nociceptors of rat dorsal root ganglion. Brain Struct Funct 220: 1885–1899. doi: 10.1007/s00429-014-0762-4 24706047 10.1007/s00429-014-0762-4

[46] Bailey AL, Ribeiro-da-Silva A (2006) Transient loss of terminals from non-peptidergic nociceptive fibers in the substantia gelatinosa of spinal cord following chronic constriction injury of the sciatic nerve. Neuroscience 138: 675–690. doi: 10.1016/j.neuroscience.2005.11.051 16413131 10.1016/j.neuroscience.2005.11.051

[47] Yucel S, De Souza A, Jr., Baskin LS (2004) Neuroanatomy of the human female lower urogenital tract. J Urol 172: 191–195. doi: 10.1097/01.ju.0000128704.51870.87 15201770 10.1097/01.ju.0000128704.51870.87

[48] Wang ZA, Mitrofanova A, Bergren SK, Abate-Shen C, Cardiff RD, Califano A, et al (2013) Lineage analysis of basal epithelial cells reveals their unexpected plasticity and supports a cell-of-origin model for prostate cancer heterogeneity. Nat Cell Biol 15: 274–283. doi: 10.1038/ncb2697 23434823 10.1038/ncb2697 PMC3743266

[49] Cheng CY, Zhou Z, Nikitin AY (2013) Detection and organ-specific ablation of neuroendocrine cells by synaptophysin locus-based BAC cassette in transgenic mice. PLoS One 8: e60905 doi: 10.1371/journal.pone.0060905 23630575 10.1371/journal.pone.0060905 PMC3632533

[50] Hofmann PG, Baez Saldana A, Fortoul Van Der Goes T, Gonzalez del Pliego M, Gutierrez Ospina G (2013) Neuroendocrine cells are present in the domestic fowl ovary. J Anat 222: 170–177. doi: 10.1111/joa.12002 23083425 10.1111/joa.12002 PMC3632222

[51] Cunha GR, Ricke W, Thomson A, Marker PC, Risbridger G, Hayward SW, et al (2004) Hormonal, cellular, and molecular regulation of normal and neoplastic prostatic development. J Steroid Biochem Mol Biol 92: 221–236. doi: 10.1016/j.jsbmb.2004.10.017 15663986 10.1016/j.jsbmb.2004.10.017

[52] Barron DA, Rowley DR (2012) The reactive stroma microenvironment and prostate cancer progression. Endocr Relat Cancer 19: R187–204. doi: 10.1530/ERC-12-0085 22930558 10.1530/ERC-12-0085 PMC3716392

[53] Cunha GR, Hayward SW, Wang YZ (2002) Role of stroma in carcinogenesis of the prostate. Differentiation 70: 473–485. doi: 10.1046/j.1432-0436.2002.700902.x 12492490 10.1046/j.1432-0436.2002.700902.x

[54] Niu Y, Altuwaijri S, Lai KP, Wu CT, Ricke WA, Messing EM, et al (2008) Androgen receptor is a tumor suppressor and proliferator in prostate cancer. Proc Natl Acad Sci U S A 105: 12182–12187. doi: 10.1073/pnas.0804700105 18723679 10.1073/pnas.0804700105 PMC2527886

[55] Tuxhorn JA, Ayala GE, Smith MJ, Smith VC, Dang TD, Rowley DR. (2002) Reactive stroma in human prostate cancer: induction of myofibroblast phenotype and extracellular matrix remodeling. Clin Cancer Res 8: 2912–2923. 12231536

[56] Gharaee-Kermani M, Kasina S, Moore BB, Thomas D, Mehra R, Macoska JA. (2012) CXC-type chemokines promote myofibroblast phenoconversion and prostatic fibrosis. PLoS One 7: e49278 doi: 10.1371/journal.pone.0049278 23173053 10.1371/journal.pone.0049278 PMC3500280

[57] Gharaee-Kermani M, Mehra R, Robinson DR, Wei JT, Macoska JA (2014) Complex cellular composition of solitary fibrous tumor of the prostate. Am J Pathol 184: 732–739. doi: 10.1016/j.ajpath.2013.11.024 24434011 10.1016/j.ajpath.2013.11.024 PMC3936322

[58] Rodriguez-Nieves JA, Macoska JA (2013) Prostatic fibrosis, lower urinary tract symptoms, and BPH. Nat Rev Urol 10: 546–550. doi: 10.1038/nrurol.2013.149 23857178 10.1038/nrurol.2013.149 PMC5625295

[59] Hennenberg M, Stief CG, Gratzke C (2014) Pharmacology of the lower urinary tract. Indian J Urol 30: 181–188. doi: 10.4103/0970-1591.126903 24744518 10.4103/0970-1591.126903 PMC3989821

[60] Nelson WG, De Marzo AM, DeWeese TL, Isaacs WB (2004) The role of inflammation in the pathogenesis of prostate cancer. J Urol 172: S6–11; discussion S11-12. 15535435 10.1097/01.ju.0000142058.99614.ff

[61] Rudick CN, Schaeffer AJ, Thumbikat P (2008) Experimental autoimmune prostatitis induces chronic pelvic pain. Am J Physiol Regul Integr Comp Physiol 294: R1268–1275. doi: 10.1152/ajpregu.00836.2007 18287220 10.1152/ajpregu.00836.2007

[62] Habermacher GM, Chason JT, Schaeffer AJ (2006) Prostatitis/chronic pelvic pain syndrome. Annu Rev Med 57: 195–206. doi: 10.1146/annurev.med.57.011205.135654 16409145 10.1146/annurev.med.57.011205.135654

[63] Torkko KC, Wilson RS, Smith EE, Kusek JW, van Bokhoven A, Lucia MS. (2015) Prostate Biopsy Markers of Inflammation are Associated with Risk of Clinical Progression of Benign Prostatic Hyperplasia: Findings from the MTOPS Study. J Urol 194: 454–461. doi: 10.1016/j.juro.2015.03.103 25828974 10.1016/j.juro.2015.03.103

[64] Kurita T, Medina RT, Mills AA, Cunha GR (2004) Role of p63 and basal cells in the prostate. Development 131: 4955–4964. doi: 10.1242/dev.01384 15371309 10.1242/dev.01384

[65] Cox HM (2016) Neuroendocrine peptide mechanisms controlling intestinal epithelial function. Current Opinion in Pharmacology 31: 50–56. doi: 10.1016/j.coph.2016.08.010 27597736 10.1016/j.coph.2016.08.010

[66] Russo G, Mischi M, Scheepens W, De la Rosette JJ, Wijkstra H (2012) Angiogenesis in prostate cancer: onset, progression and imaging. BJU Int 110: E794–808. doi: 10.1111/j.1464-410X.2012.11444.x 22958524 10.1111/j.1464-410X.2012.11444.x

[67] Deering RE, Bigler SA, Brown M, Brawer MK (1995) Microvascularity in benign prostatic hyperplasia. Prostate 26: 111–115. 7534916 10.1002/pros.2990260302

[68] Kojima M, Watanabe H, Watanabe M, Okihara K, Naya Y, Ukimura O. (1997) Preliminary results of power Doppler imaging in benign prostatic hyperplasia. Ultrasound Med Biol 23: 1305–1309. 9428128 10.1016/s0301-5629(97)00141-5

[69] Foley SJ, Bailey DM (2000) Microvessel density in prostatic hyperplasia. BJU Int 85: 70–73. 10619949 10.1046/j.1464-410x.2000.00322.x

[70] Sun QZ, Guan TY, Qi JG, Cao JY, Wu G, Yang N, et al (2010) [Histological characteristics of the prostate in men who receive re-TURP for benign prostatic hyperplasia and their clinical significance]. Zhonghua Nan Ke Xue 16: 118–122. 20369693

[71] Gerhardt H, Betsholtz C (2003) Endothelial-pericyte interactions in angiogenesis. Cell and Tissue Research 314: 15–23. doi: 10.1007/s00441-003-0745-x 12883993 10.1007/s00441-003-0745-x

[72] Brozovich FV, Nicholson CJ, Degen CV, Gao YZ, Aggarwal M, Morgan KG. (2016) Mechanisms of Vascular Smooth Muscle Contraction and the Basis for Pharmacologic Treatment of Smooth Muscle Disorders. Pharmacol Rev 68: 476–532. doi: 10.1124/pr.115.010652 27037223 10.1124/pr.115.010652 PMC4819215

[73] Tian H, Ketova T, Hardy D, Xu X, Gao X, Zijlstra A, et al (2017) Endoglin Mediates Vascular Maturation by Promoting Vascular Smooth Muscle Cell Migration and Spreading. Arterioscler Thromb Vasc Biol 37: 1115–1126. doi: 10.1161/ATVBAHA.116.308859 28450296 10.1161/ATVBAHA.116.308859 PMC5444426

[74] Yin GN, Das ND, Choi MJ, Song KM, Kwon MH, Ock, J, et al. (2015) The pericyte as a cellular regulator of penile erection and a novel therapeutic target for erectile dysfunction. Scientific Reports 5. 10.1038/srep10891 PMC4456662 26044953

[75] Ozawa MG, Yao VJ, Chanthery YH, Troncoso P, Uemura A, Varner AS, et al (2005) Angiogenesis with pericyte abnormalities in a transgenic model of prostate carcinoma. Cancer 104: 2104–2115. doi: 10.1002/cncr.21436 16208706 10.1002/cncr.21436

[76] Humphreys BD, Lin SL, Kobayashi A, Hudson TE, Nowlin BT, Bonventre JV, et al (2010) Fate tracing reveals the pericyte and not epithelial origin of myofibroblasts in kidney fibrosis. Am J Pathol 176: 85–97. doi: 10.2353/ajpath.2010.090517 20008127 10.2353/ajpath.2010.090517 PMC2797872

[77] LeBleu VS, Taduri G, O'Connell J, Teng YQ, Cooke VG, Woda C, et al (2013) Origin and function of myofibroblasts in kidney fibrosis. Nature Medicine 19: 1047–1054. doi: 10.1038/nm.3218 23817022 10.1038/nm.3218 PMC4067127

[78] Birbrair A, Zhang T, Files DC, Mannava S, Smith T, Wang ZM, et al (2014) Type-1 pericytes accumulate after tissue injury and produce collagen in an organ-dependent manner. Stem Cell Res Ther 5: 122 doi: 10.1186/scrt512 25376879 10.1186/scrt512 PMC4445991

[79] Armulik A, Genove G, Betsholtz C (2011) Pericytes: developmental, physiological, and pathological perspectives, problems, and promises. Dev Cell 21: 193–215. doi: 10.1016/j.devcel.2011.07.001 21839917 10.1016/j.devcel.2011.07.001

[80] Leystra AA, Deming DA, Zahm CD, Farhoud M, Olson TJ, Hadac JN, et al (2012) Mice expressing activated PI3K rapidly develop advanced colon cancer. Cancer Res 72: 2931–2936. doi: 10.1158/0008-5472.CAN-11-4097 22525701 10.1158/0008-5472.CAN-11-4097 PMC3645915

[81] McMenamin ME, Soung P, Perera S, Kaplan I, Loda M, Sellers WR. (1999) Loss of PTEN expression in paraffin-embedded primary prostate cancer correlates with high Gleason score and advanced stage. Cancer Res 59: 4291–4296. 10485474

[82] Mehta V, Schmitz CT, Keil KP, Joshi PS, Abler LL, Lin TM, et al (2013) Beta-catenin (CTNNB1) induces Bmp expression in urogenital sinus epithelium and participates in prostatic bud initiation and patterning. Dev Biol 376: 125–135. doi: 10.1016/j.ydbio.2013.01.034 23396188 10.1016/j.ydbio.2013.01.034 PMC3602957

[83] Domen J, Wagers A, Weissman IL (2016) Bone Marrow (Hematopoietic) Stem Cells. National Institutes of Health, U.S. Department of Health and Human Services.

[84] Seemayer TA, Lagace R, Schurch W, Tremblay G (1979) Myofibroblasts in the stroma of invasive and metastatic carcinoma: a possible host response to neoplasia. Am J Surg Pathol 3: 525–533. 534389 10.1097/00000478-197912000-00005

[85] Tuxhorn JA, Ayala GE, Rowley DR (2001) Reactive stroma in prostate cancer progression. J Urol 166: 2472–2483. 11696814

[86] Walkin L, Herrick SE, Summers A, Brenchley PE, Hoff CM, Korstanje R, et al (2013) The role of mouse strain differences in the susceptibility to fibrosis: a systematic review. Fibrogenesis Tissue Repair 6: 18 doi: 10.1186/1755-1536-6-18 24294831 10.1186/1755-1536-6-18 PMC3849643

[87] Shinagawa K, Kojima M (2003) Mouse model of airway remodeling: strain differences. Am J Respir Crit Care Med 168: 959–967. doi: 10.1164/rccm.200210-1188OC 12857720 10.1164/rccm.200210-1188OC

[88] Marques SM, Campos PP, Castro PR, Cardoso CC, Ferreira MA, Andrade SP. (2011) Genetic background determines mouse strain differences in inflammatory angiogenesis. Microvasc Res 82: 246–252. doi: 10.1016/j.mvr.2011.08.011 21907724 10.1016/j.mvr.2011.08.011

