# Supplementary figures and images for: An immunohistochemical identification key for cell types in adult mouse prostatic and urethral tissue sections
Source: PLoS One. 2017 Nov 16;12(11):e0188413. doi: 10.1371/journal.pone.0188413 (PMC5690684; doi:10.1371/journal.pone.0188413)

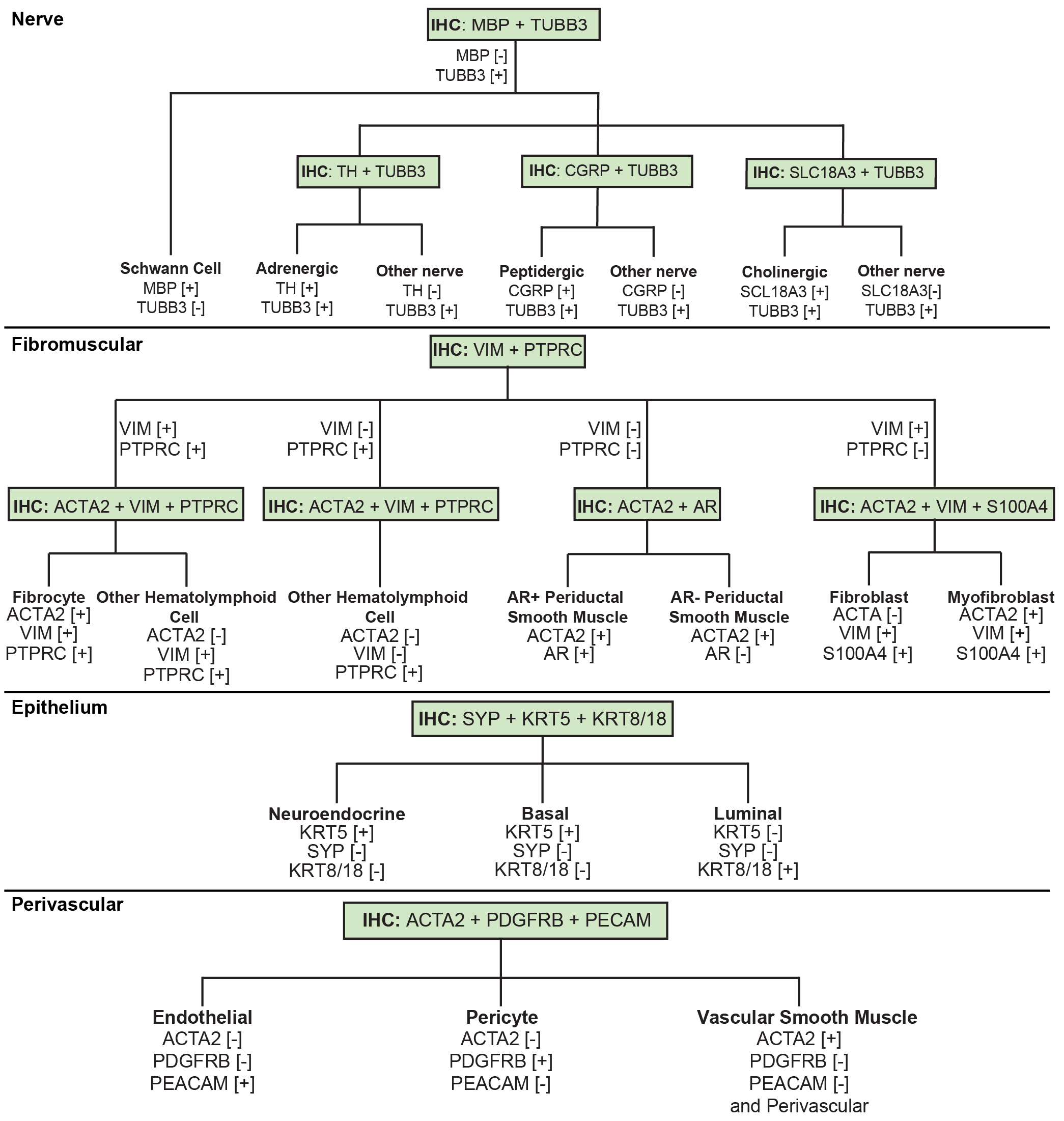

Supplement: S1 Fig — (TIF) [file pone.0188413.s001.tif]

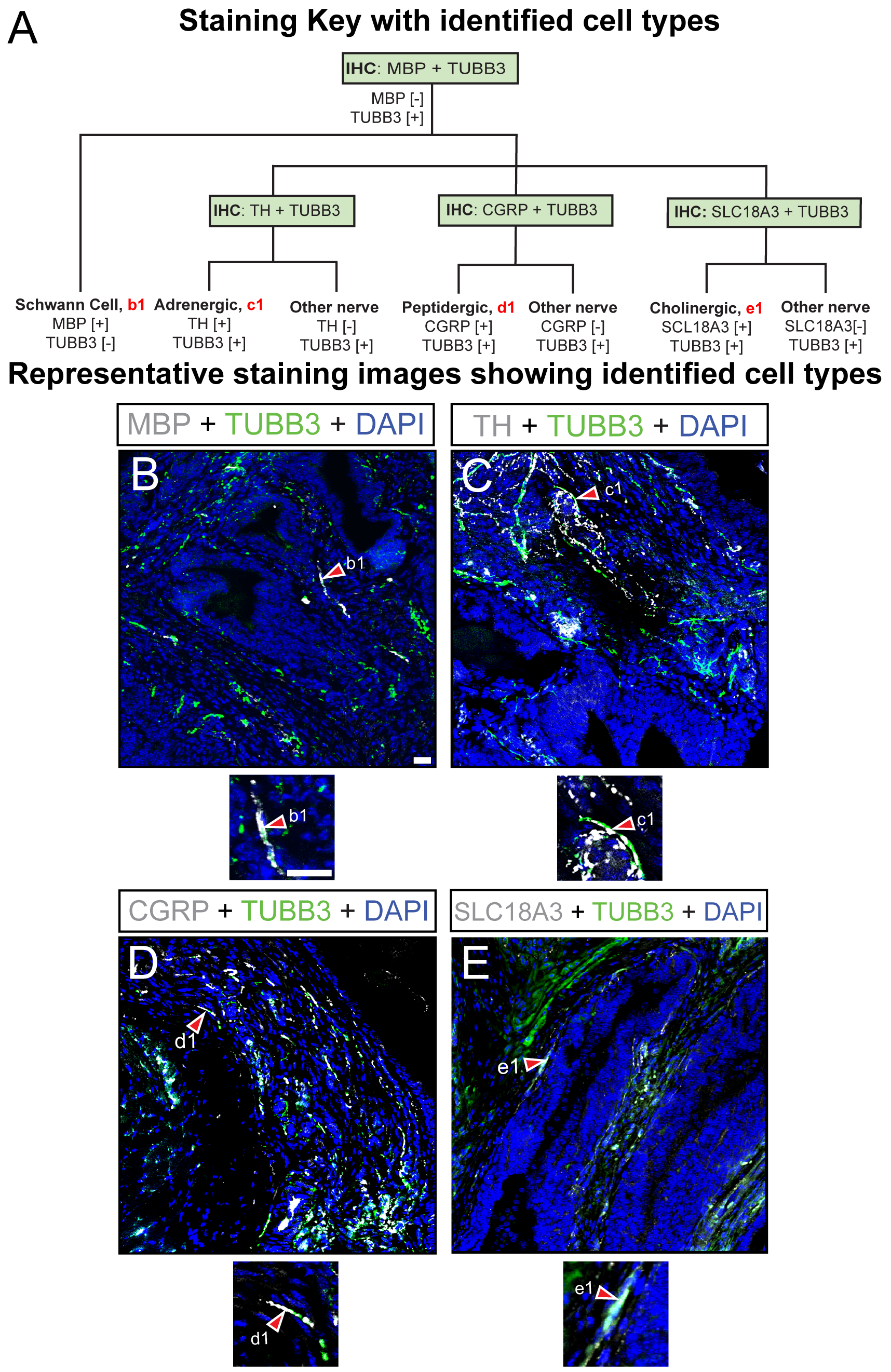

Supplement: S2 Fig — (A) Paraffin embedded adult mouse prostatic urethra sections (5 μm thickness) were stained with DAPI and antibodies against (B) MBP and TUBB3 (C) TH and TUBB3, CGRP and TUBB3, or (E) SLC18A3 and TUBB3. Identified cells include (b1) MBP1+; TUBB3-; Schwann cells, (c1) TH+;TUBB3+ adrenergic fibers (d1) CGRP+;TUBB3+ sensory fibers, (e1) SLC18A3+;TUBB3+ cholinergic fibers. Images are representative of three mice. Abbreviations are: MBP, myelin basic protein; CGRP, calcitonin-gene-related peptide; SLC18A3, solute carrier family 18 member 3; TH, tyrosine hydroxylase; DAPI, 2-(4-amidinophenyl)-1H -indole-6-carboxamidine; Scale bar is 25 μm. (TIF) [file pone.0188413.s002.tif]

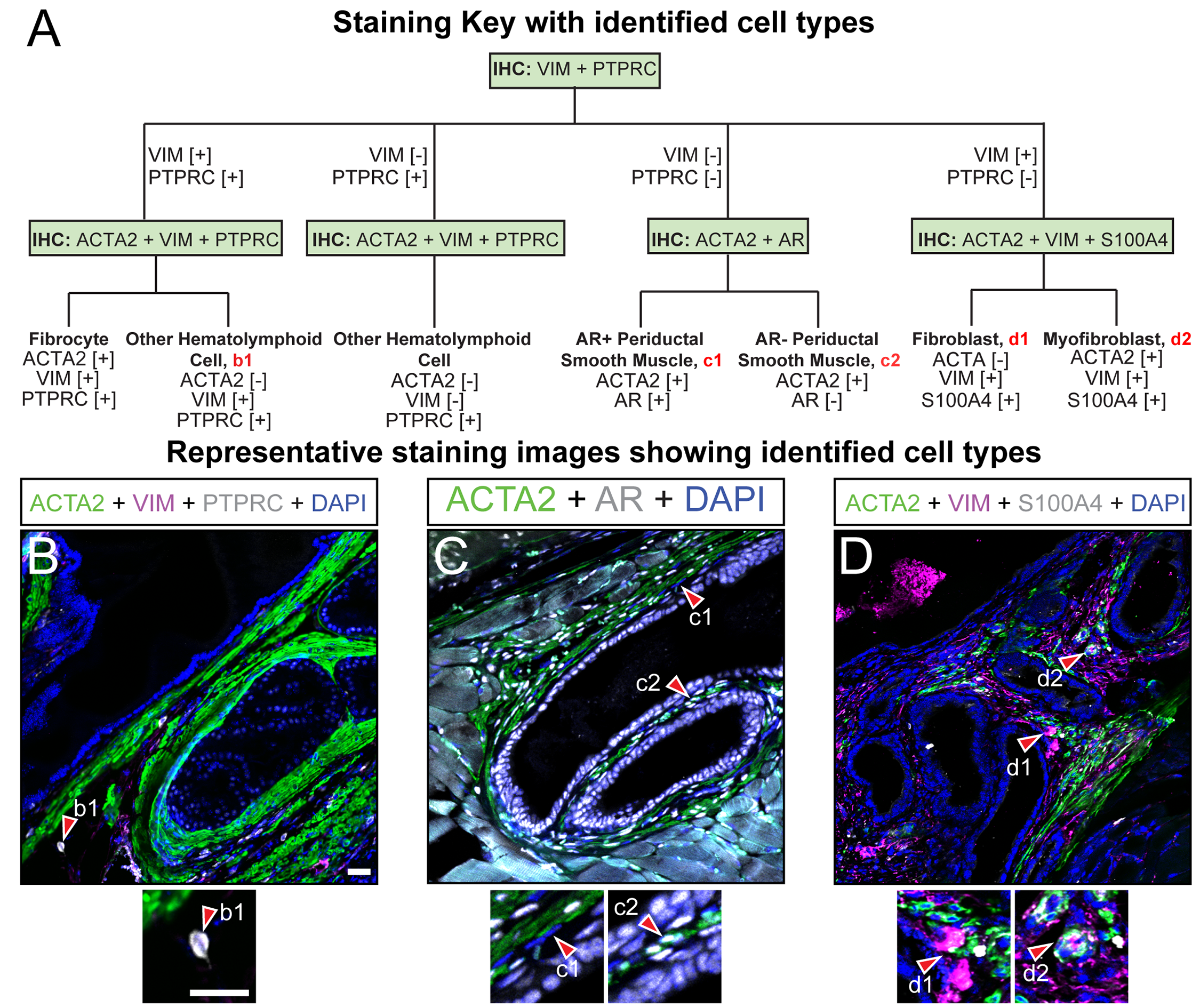

Supplement: S3 Fig — (A) Paraffin embedded adult mouse prostatic urethra sections (5 μm thickness) were stained with DAPI and antibodies against (B) ACTA2, VIM, and PTPRC, (C) ACTA2 and AR, or (D) ACTA2, VIM, and S100A4. The identified cells include (b1) ACTA2-;VIM+;PTPRC+ hematolymphoid cells, (c1) ACTA2+;AR+ smooth muscle myoctyes, (c2) ACTA2+;AR- smooth muscle myocytes, (d1) ACTA2-;VIM+;S100A4+ fibroblasts, and (d2) ACTA2+;VIM+;S100A4+ myofibroblasts Images are representative of n = 3 mice. Abbreviations: PTPRC, CD45; ACTA2, actin alpha 2; VIM, vimentin; AR, androgen receptor; S100A4, fibroblast specific protein 1; DAPI, 2-(4-amidinophenyl)-1H -indole-6-carboxamidine; Scale bar is 25 μm. (TIF) [file pone.0188413.s003.tif]

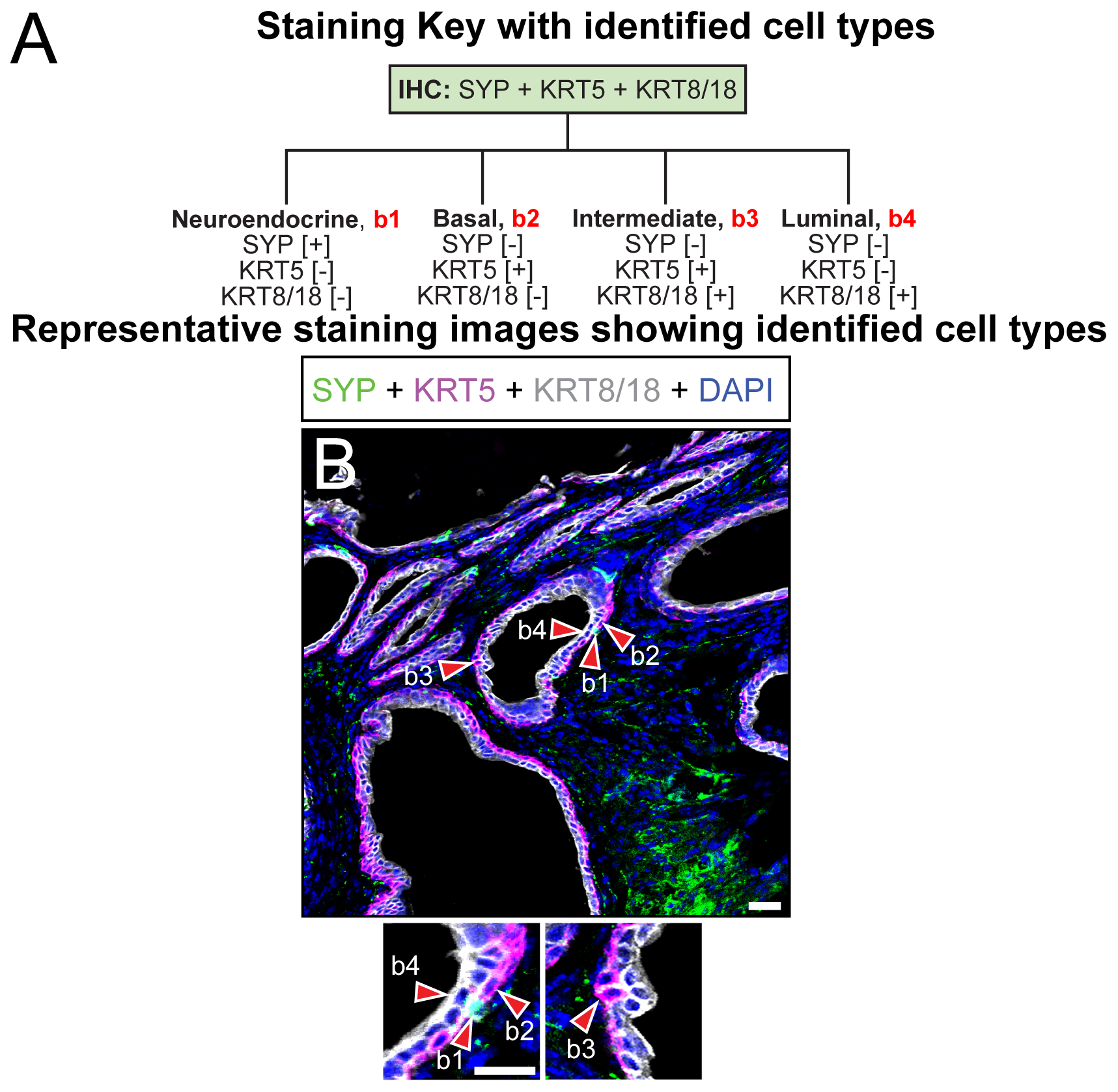

Supplement: S4 Fig — (A) Paraffin embedded adult mouse prostatic urethra sections (5 μm thickness) were stained with DAPI and antibodies against (B) KRT5, SYP, and KRT8/18. Identified cells include (b1) KRT5-;SYP+;KRT8/18- neuroendocrine cells, (b2) KRT5+;SYP-;KRT8/18- basal epithelial cells, and (b3) KRT5-;SYP-;KRT8/18+ luminal epithelial cells. Images are representative of three mice. Abbreviations: SYP, synaptophysin; KRT5, keratin 5; KRT8/18, keratin 8/18; DAPI, 2-(4-amidinophenyl)-1H -indole-6-carboxamidine; Scale bar is 25 μm. (TIF) [file pone.0188413.s004.tif]

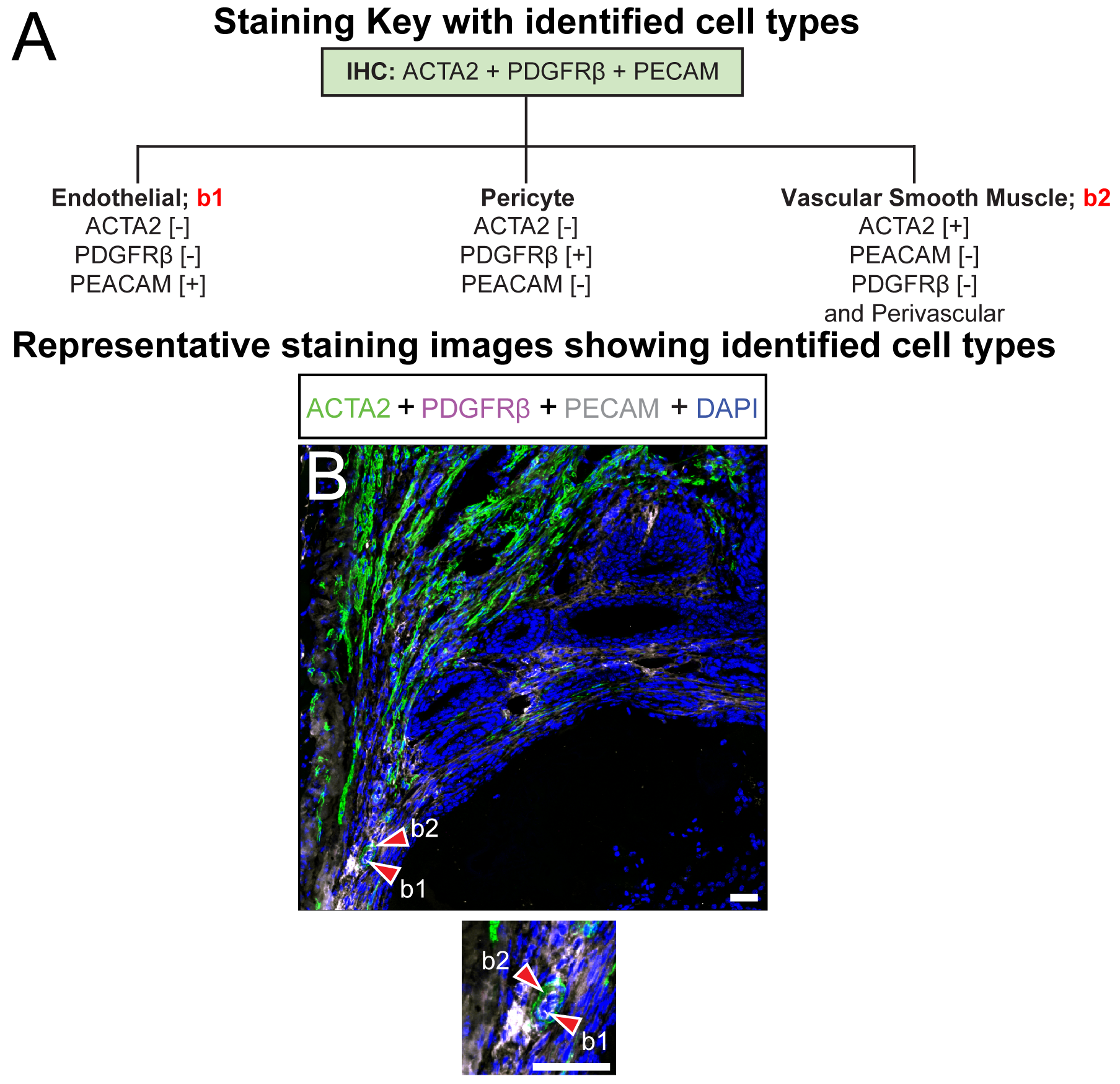

Supplement: S5 Fig — (A) Paraffin embedded adult mouse prostatic urethra sections (15 μm thickness) were stained with DAPI and antibodies against (B, C) ACTA2, PDGFRB, and PECAM. Identified cells include (b1, c1) ACTA2-;PDGFRB-;PECAM+ endothelial cells, (b2) ACTA2-;PDGFRB+;PECAM- pericytes, and (b3, c2) ACTA2+;PDGFRB-;PECAM- vascular smooth muscle cells. Images are representative of three mice. Abbreviations: ACTA2, actin alpha 2; PDGFRB, platelet derived growth factor receptor beta; PECAM, platelet endothelial cell adhesion molecule; DAPI, 2-(4-amidinophenyl)-1H -indole-6-carboxamidine; Scale bar is 25 μm. (TIF) [file pone.0188413.s005.tif]

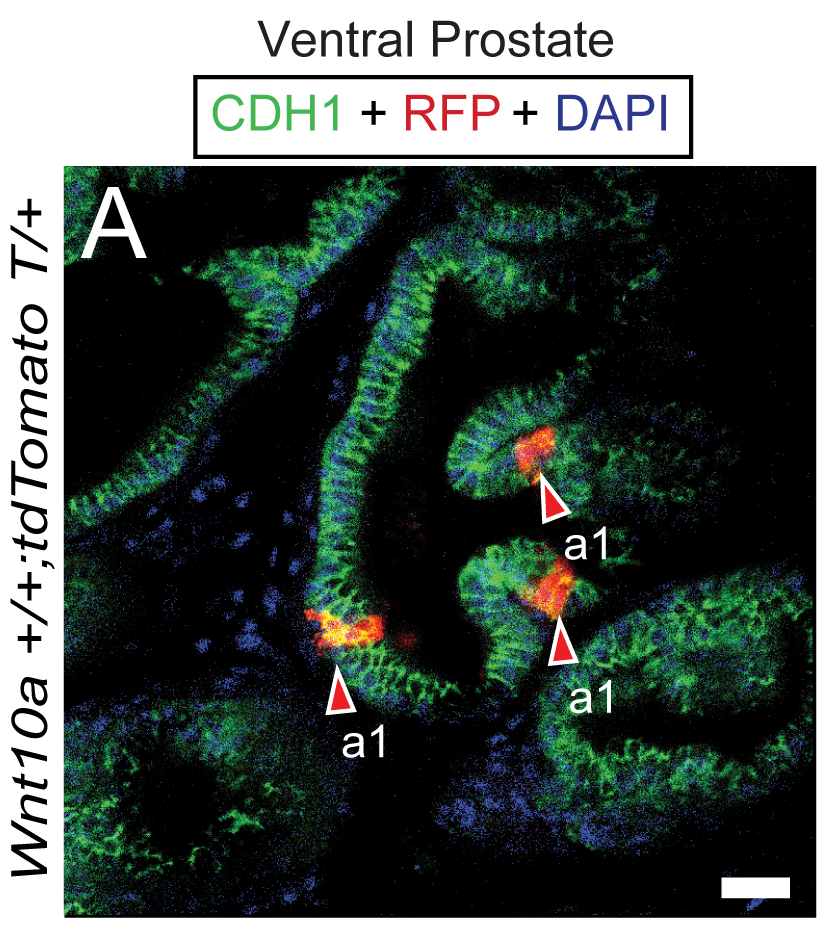

Supplement: S6 Fig — Wnt10aCreER/+T;tdtomatofl/fl and Wnt10a+/-;tdtomatofl/fl (control) male mice were given a single intraperitoneal injection of tamoxifen (100 mg/kg) on postnatal day 3 and aged to two months. Prostates were sectioned (5 μm thickness), and stained with DAPI and antibodies against (A) CDH1, and RFP/tdtomato. The tdtomato lineage label was identified in CDH1+ luminal epithelial cells. Image is representative of three mice. Abbreviations: CDH1, E Cadherin; RFP, red fluorescent protein; DAPI, 2-(4-amidinophenyl)-1H -indole-6-carboxamidine; Scale bar is 25 μm. (TIF) [file pone.0188413.s006.tif]

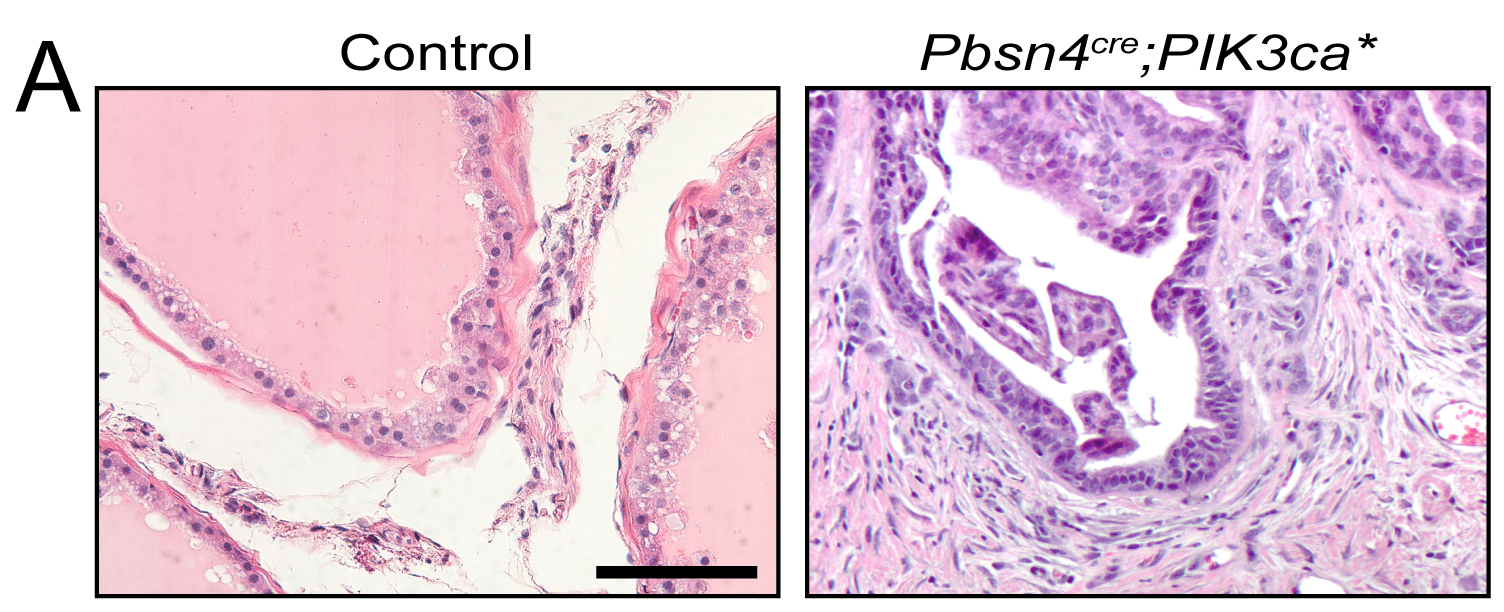

Supplement: S7 Fig — (A) Paraffin embedded adult mouse prostate sections (5 μm thickness) generated from mice with genetic activation of the PIK3/AKT signaling cascade in prostate epithelial cells (Pbsn4cre;PIK3ca*) and were stained with hematoxylin and eosin to reveal a marked increase in the fibromuscular stroma of the prostate. Scale bar is 100 μm. (TIF) [file pone.0188413.s007.tif]
