# Supplementary material for: An immunohistochemical identification key for cell types in adult mouse prostatic and urethral tissue sections
Source: PLoS One. 2017 Nov 16;12(11):e0188413. doi: 10.1371/journal.pone.0188413 (PMC5690684; doi:10.1371/journal.pone.0188413)
Supplement: S1 Table — (DOCX) [file pone.0188413.s008.docx]

**S1 Table. Antibodies used for immunostaining.**

| **Name(s)** | **Symbol** | **Species** | **Antibody Registry (RRID)** | **Supplier** | **Catalog Number** | **Dilution** | **Un-masking** | **Fig #** |
| --- | --- | --- | --- | --- | --- | --- | --- | --- |
| Actin alpha 2 | ACTA2 | Goat | AB_10980764 | Thermo Fisher Sci. | PA5-18292 | 1:100 | Citrate | 1,3,6,S2,S3 |
| Actin alpha 2 | ACTA2 | Mouse | AB_442134 | Leica | NCL-L-SMA | 1:250 | Citrate | 5,S5 |
| Androgen Receptor | AR | Rabbit | AB_1563391 | Santa Cruz Biotech. | SC-816 | 1:250 | Citrate | 3,6,S3 |
| Calcitonin Gene Related Peptide | CGRP | Rabbit | AB_259091 | Sigma | C8198 | 1:200 | Citrate | 2,S2 |
| Chondroitin sulfate proteoglycan 4 | CSPG4 | Rabbit | AB_91789 | Abcam | AB5320 | 1:250 | Tris EDTA | 5,S5 |
| E-Cadherin | CDH1 | Mouse | AB_397580 | BD Biosciences | 610181 | 1:250 | Citrate | 1,S6 |
| Keratin 5 | KRT5 | Chick | AB_2565054 | Biolegend | 905901 | 1:100 | Citrate | 4,7,S4 |
| Keratin 8/18 | KRT8/18 | Rat | AB_531826 | Developmental Studies Hybridoma Bank | Troma I | 1:300 | Citrate | 4,7,S4 |
| Myelin basic protein | MBP | Mouse | AB_956157 | Abcam | AB62631 | 1:400 | Citrate | 2,  S2 |
| Platelet and endothelial cell adhesion molecule | PECAM | Mouse | N/A | Santa Cruz Biotech. | SC-376764 | 1:200 | Tris EDTA | 5,S5 |
| Platelet derived growth factor receptor beta | PDGFRB | Rabbit | AB_2162497 | Cell Signaling Tech. | 3169S | 1:100 | Tris EDTA | 5,S5 |
| Protein tyrosine phosphatase, receptor type C | PTPRC | Rabbit | AB_442810 | Abcam | AB10558 | 1:500 | Citrate | 3,6,S3 |
| Red Fluorescent Protein | RFP | Rabbit | AB_2209751 | Rockland | 600-401-379 | 1:500 | Citrate | 7,S6 |
| S100 calcium binding protein A4 | S100A4 | Rabbit | AB_2183775 | Abcam | AB27957 | 1:200 | Citrate | 3,6,S3 |
| solute carrier family 18 (vesicular monoamine), member 3 | SLC18A3 | Goat | AB_2630394 | EMD Millipore | ABN100 | 1:100 | Citrate | 2, S2 |
| Synaptophysin | SYP | Mouse | AB_399360 | BD Biosciences | 611880 | 1:200 | Citrate | 4,7,S4 |
| Tubulin beta 3 | TUBB3 | Chick | AB_10899689 | Abcam | AB107216 | 1:100 | Citrate | 2,S2 |
| Tubulin beta 3 | TUBB3 | Mouse | AB_1070721 | Invitrogen | MA1-19187 | 1:400 | Citrate | 2,S2 |
| Tyrosine Hydroxylase | TH | Rabbit | AB_390204 | Abcam | AB152 | 1:100 | Citrate | 2,S2 |
| Vimentin | VIM | Mouse | AB_445527 | Abcam | AB20346 | 1:250 | Citrate | 3,6,S3 |
| Anti-mouse Alexa Fluor 488 | - | Donkey | AB_2340862 | Jackson Immuno Res. | 715-605-150 | 1:250 | - | 1,2,4,7,S2,S4 |
| Anti-chicken RRX | - | Donkey | AB_2340371 | Jackson Immuno Res. | 703-295-155 | 1:250 | - | 2,4,7,S2,S4 |
| Anti-mouse Alexa Fluor 488 | - | Goat | AB_2338869 | Jackson Immuno Res. | 115-547-003 | 1:250 | - | 2,3,6S2,S3 |
| Anti-rabbit Alexa Fluor 594 | - | Goat | N/A | Jackson Immuno Res. | 111-516-045 | 1:250 | - | 1,2,3,6,S2,S3 |
| Anti-mouse Alexa Fluor 594 | - | Donkey | AB_2340854 | Jackson Immuno Res. | 715-585-150 | 1:250 | - | 2,S2 |
| Anti-rat Brilliant Violet 480 | - | Donkey | AB_2651112 | Jackson Immuno Res. | 712-685-150 | 1:250 | - | **7** |
| Anti-mouse Alexa Fluor 647 | - | Donkey | AB_2340846 | Jackson Immuno Res. | 715-545-150 | 1:250 | - | 3,5,6,S3,S5 |
| Anti-rabbit Alexa Fluor 647 | - | Goat | AB_2338083 | Jackson Immuno Res. | 111-606-144 | 1:250 | - | 7 |
| Anti-rabbit Alexa Fluor 546 | - | Donkey | AB_2534016 | Thermo Fisher Scientific | A10040 | 1:250 | - | 3,5,6S3,S5 |
| Anti-goat Alexa Fluor 488 | - | Donkey | AB_2340428 | Jackson Immuno Res. | 705-545-003 | 1:250 | - | 2,3,5,6,S2,S3,S5 |
